# Synthesis, Anticancer and Antibacterial Activity of Salinomycin *N*-Benzyl Amides

**DOI:** 10.3390/molecules191219435

**Published:** 2014-11-25

**Authors:** Michał Antoszczak, Ewa Maj, Agnieszka Napiórkowska, Joanna Stefańska, Ewa Augustynowicz-Kopeć, Joanna Wietrzyk, Jan Janczak, Bogumil Brzezinski, Adam Huczyński

**Affiliations:** 1 Faculty of Chemistry, Adam Mickiewicz University, Umultowska 89 b, 61-614 Poznań, Poland; E-Mails: michant@amu.edu.pl (M.A.); bbrzez@amu.edu.pl (B.B.); 2 Ludwik Hirszfeld Institute of Immunology and Experimental Therapy, Polish Academy of Sciences, Rudolfa Weigla 12, 53-114 Wrocław, Poland; E-Mails: ewa.maj@iitd.pan.wroc.pl (E.M.); wietrzyk@iitd.pan.wroc.pl (J.W.); 3 Department of Microbiology, Institute of Tuberculosis and Pulmonary Diseases, Płocka 26, 01-138 Warsaw, Poland; E-Mails: araceli@op.pl (A.N.); e.kopec@igichp.edu.pl (E.A.-K.); 4 Department of Pharmaceutical Microbiology, University of Warsaw, Oczki 3, 02-007 Warsaw, Poland; E-Mail: jstefanska@wum.edu.pl; 5 Institute of Low Temperature and Structure Research, Polish Academy of Sciences, P.O. Box 1410, 50-950 Wrocław, Poland; E-Mail: J.Janczak@int.pan.wroc.pl

**Keywords:** anticancer activity, antibacterial activity, antitubercular activity, SAR studies, ionophores

## Abstract

A series of 12 novel monosubstituted *N*-benzyl amides of salinomycin (**SAL**) was synthesized for the first time and characterized by NMR and FT-IR spectroscopic methods. Molecular structures of three salinomycin derivatives in the solid state were determined using single crystal X-ray method. All compounds obtained were screened for their antiproliferative activity against various human cancer cell lines as well as against the most problematic bacteria strains such as methicillin-resistant *Staphylococcus aureus* (MRSA) and *Staphylococcus epidermidis* (MRSE), and *Mycobacterium tuberculosis.* Novel salinomycin derivatives exhibited potent anticancer activity against drug-resistant cell lines. Additionally, two *N*-benzyl amides of salinomycin revealed interesting antibacterial activity. The most active were *N-*benzyl amides of **SAL** substituted at -*ortho* position and the least anticancer active derivatives were those substituted at the -*para* position.

## 1. Introduction

**Figure 1:**
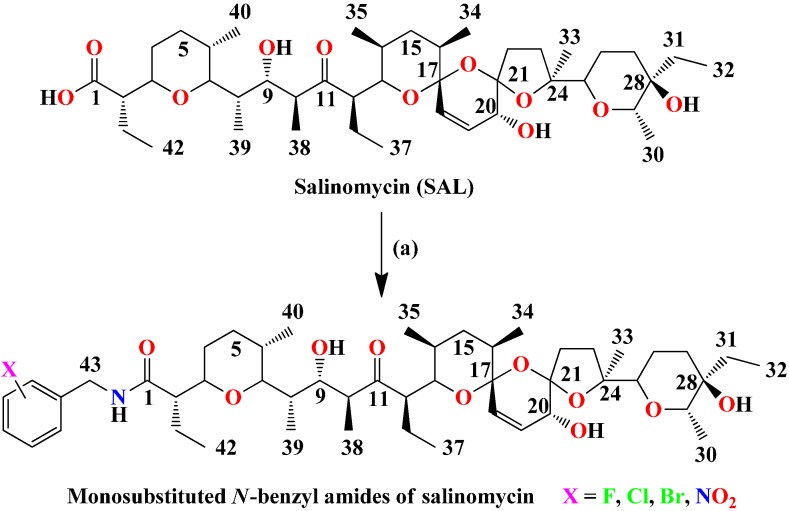
Synthesis of monosubstitued *N*-benzyl amides of salinomycin.

Salinomycin (**SAL**) (Figure 1) is an antibiotic that belongs to a large group of polyether ionophores [1]. It has been found that **SAL** exhibits high antimicrobial activity against Gram-positive bacteria, including mycobacteria and some filamentous fungi [2,3]. Furthermore, salinomycin sodium salt is commonly used in veterinary medicine as a non-hormonal growth promoting as well as anti-coccidiostat agent [4,5]. Where is reference 6, please cite reference in order.

*Reagents and conditions*: (a) DCC, HOBt, R-NH_2_ (appropriate mono-substituted benzyl amine), Et_3_N (if the substrate is an amine hydrochloride), CH_2_Cl_2_/THF (3/1), 0 °C—1 h, then rt—24 h. Time for completion of the reaction at rt as indicated by TLC.

Especial attention has been paid to **SAL** since 2009, when it was announced that this compound was nearly 100-fold more effective towards the breast cancer stem cells (CSCs) than the commonly used cytostatic drug—*Taxol* [6]. Extensive research work has been undertaken to explain the unusual anticancer properties of this antibiotic. It has been proved that **SAL** is capable of inducing programmed cell death (apoptosis) of human cancer cells of various tissues exhibiting multidrug resistance (MDR), for example in the case of leukemic CSCs by expression of ATP-binding cassette transporters (ABC) [7]. Moreover, it has been reported that **SAL** strongly inhibits a proximal Wnt/β-catenin signalling and blocks phosphorylation of the Wnt-LRP6 co-receptor, which leads to its degradation and consequently to apoptosis of cancer cells in chronic lymphocytic leukemia [8]. The ability of **SAL** to reduce the subpopulation of colon adenocarcinoma CSCs and the considerable activity against human colon cancer cells have been also observed. These cells were more affected by **SAL** than by oxaliplatin, a cytostatic drug commonly used in the anticancer chemotherapy of colorectal cancer [9]. It has been established that **SAL** causes concentration- and time-dependent reduction in viability of LNM-35 and A-549 human lung cancer cell lines through a caspase cell death pathway, as well as induces a marked increase in the expression of the pro-apoptotic protein NAG-1, which leads to the inhibition of lung cancer cell invasion [10].

**SAL** blocks the growth and migration of chemo-resistant prostate cancer cells and also causes accumulation of reactive oxygen species (ROS), which leads to depolarization of the mitochondrial membrane and cell apoptosis [11]. The inhibitory effect of **SAL** on the proliferation, migration as well as invasion of human endometrial and nasopharyngeal CSCs has been also documented [12,13]. In addition, the sensitizing effect of **SAL** has been evidenced upon treatment with cytostatic agents, such as *Taxol*, *Docetaxel*, vinblastine and colchicine [14,15,16]. Synergistic anticancer effect of **SAL** in combination with gemcitabine against human pancreatic cancer cells has been observed [17].

Since 2012 **SAL** was approved for testing in the screening studies on a small group of patients with invasive carcinoma of the head, neck, breast and ovary. Patients were treated with 200–250 µg/kg of **SAL** intravenously every second day for three weeks. Two cases were described in literature in detail, in which the therapy of patients with **SAL** resulted in inhibition of disease progress over an extended period of time. Acute side effects were rare and the serious long-term adverse side effects were not observed [18].

An important direction of research is the chemical modification of **SAL**, which can lead to derivatives with significantly lower toxicity and with better biological activity than that of unmodified antibiotic, due to favorable changes of ionophoretic properties and the transport of metal cations through the lipid membranes. In our previous works we have proved that chemically modified polyether antibiotics, such as amides and esters, transport metal cations by an electrogenic or biomimetic mechanism, wherein the unmodified ionophores transport metal cations only by an electroneutral mechanism [19,20].

Until now, the synthesis, chemical structure and biological properties of various amides, including diamides, esters and *O*-acylated derivatives of **SAL** have been described [21,22,23,24,25,26]. Results of the tests have clearly shown that all **SAL** derivatives are more or less biologically active in the specified concentration range.

In the preliminary structure-activity relationship (SAR) studies we have demonstrated that the most potent anticancer and antimicrobial compounds among **SAL** amides are those that contain fluorine atoms in their structure, with or without aliphatic-aromatic, especially *N-*benzyl, substituents. The present paper describes an efficient method for the synthesis of 12 new mono-substituted *N-*benzyl amides of **SAL** with fluorine, chlorine and bromine atoms as well as nitro group in -*ortho*, -*meta* and -*para* positions (Figure 1).

The structures of all **SAL** mono-substituted *N-*benzyl amides were characterized using FT-IR and NMR methods and three of them were also evaluated using single crystal X-ray diffraction method. Additionally, their ability to complex monovalent and divalent metal cations was tested using the electrospray ionisation mass spectrometry (ESI MS). The *in vitro* antibacterial activity, especially against MRSA and MRSE, tuberculostatic activity against one standard and two “wild” *M. tuberculosis* strains as well as the anticancer activity of these compounds against drug-sensitive and drug-resistant human cancer cell lines were determined and discussed.

## 2. Results and Discussion

### 2.1. Synthesis and Spectroscopic Characterization of Salinomycin N-Benzyl Amides

Salinomycin sodium salt (**SAL-Na**) was isolated from widely available veterinary premix—SACOX^®^ following the procedures described previously [22]. The structure and homogeneity of isolated **SAL-Na** was confirmed using spectroscopic methods. **SAL** was obtained from **SAL-Na** by the extraction with sulfuric acid solution (pH = 1.5) in dichloromethane [22]. The 12 novel mono-substituted *N-*benzyl amides of **SAL** containing fluorine, chlorine and bromine atoms as well as nitro group at the -*ortho* (**F-*o***, **Cl-*o***, **Br-*o***, **NO_2_-*o***), -*meta* (**F-*m***, **Cl-*m***, **Br-*m***, **NO_2_-*m***) and -*para* (**F-*p***, **Cl-*p***, **Br-*p***, **NO_2_-*p***) positions were synthesized in the reaction between **SAL** and appropriate mono-substituted benzyl-amine in the presence of DCC (*N,N′*-dicyclohexylcarbodiimide) as a coupling agent and HOBt (1-hydroxybenzotriazole) as an activator [22]. Additionally, when using mono-substituted benzyl-amine hydrochloride, it was necessary to add an equimolar amount of triethylamine.

All **SAL** mono-substituted *N-*benzyl amides can be easily isolated in pure form following the purification by Dry Vacuum Column Chromatography [27], using dichloromethane/THF (100:3) mixture as mobile phase. This method was efficient and gave mono-substituted *N-*benzyl amides in high yields of up to 67%–84% (Table 1).

**Table 1:** The yields of the synthesis and the analytical signals in the ^1^H and ^13^C NMR spectra as well as the position of characteristic amide I and amide II bands in the FT-IR spectra of mono-substituted *N-*benzyl amides of **SAL**. ^1^H, ^13^C and 2D NMR spectra of selected **Br-*o*** amide are included in the Supplementary material (Figures S1–S5).

Compound	Yield (%)	Analytical NMR Signals (ppm) in CD_2_Cl_2_				Characteristic FT-IR Bands (cm^−1^)	
		^13^C(1)=O	^1^H-N_(amide)_	^13^C(43)	^1^H-C(43)	Amide I	Amide II
**F-*o***	84	175.7	6.92	40.7	4.75 (ddd, *J =* 20.9, 15.6, 5.9 Hz)	1660	1528
**F-*m***	72	175.8	6.89	42.5	4.80 (ddd, *J =* 20.6, 15.6, 6.1 Hz)	1658	1531
**F-*p***	79	175.7	6.97	42.3	4.74 (ddd, *J =* 20.3, 15.3, 6.0 Hz)	1651	1532
**Cl-*o***	73	175.3	6.90	41.3	4.82 (ddd, *J =* 21.2, 15.8, 5.9 Hz)	1660	1528
**Cl-*m***	80	175.8	7.15	42.4	4.81 (ddd, *J =* 20.5, 15.6, 6.1 Hz)	1648	1530
**Cl-*p***	77	175.7	7.03	42.4	4.75 (ddd, *J =* 20.4, 15.4, 6.1 Hz)	1658	1526
**Br-*o***	71	175.8	6.98	44.0	4.73 (dq, *J =* 16.2, 5.9 Hz)	1662	1528
**Br-*m***	75	175.8	6.98	44.0	4.73 (dq, *J =* 16.0, 5.8 Hz)	1658	1528
**Br-*p***	67	175.8	7.03	42.4	4.73 (ddd, *J =* 20.4, 15.5, 6.0 Hz)	1658	1525
**NO_2_-*o***	81	176.1	7.18	41.3	5.00 (dq, *J =* 16.4, 6.1 Hz)	1665	1530
**NO_2_-*m***	69	176.2	7.37	42.4	4.94 (ddd, *J =* 21.0, 15.8, 6.0 Hz)	1657	1532
**NO_2_-*p***	76	176.2	7.30	42.7	4.93 (ddd, *J =* 21.3, 16.2, 6.1 Hz)	1660	1523

**Figure 2:**
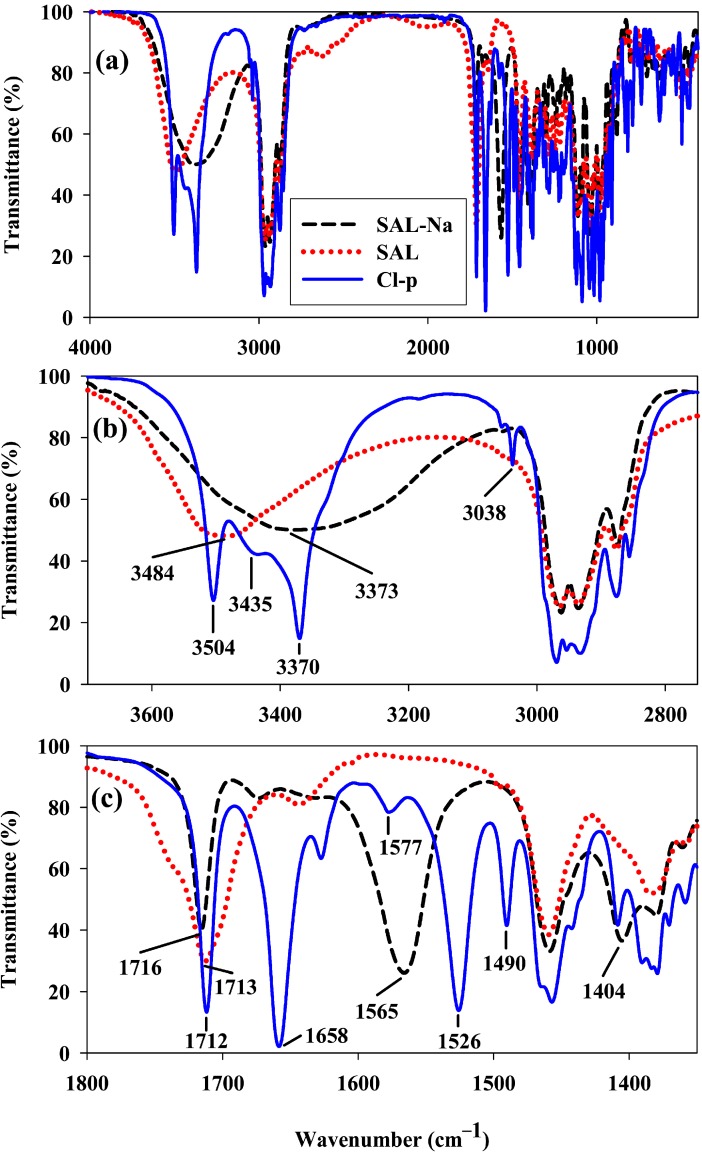
FT-IR spectra of salinomycin sodium salt (**SAL-Na**), salinomycin (**SAL**) and *p*-chlorobenzyl amide of salinomycin (**Cl-*p***) made for KBr tablet; (**a**) 4000–400 cm^−1^; (**b**) 3700–2750 cm^−1^; (**c**) 1800–1350 cm^−1^.

The purity and structures of these compounds were determined on the basis of elemental, FT-IR and NMR analysis. The ^1^H and ^13^C NMR signals were assigned using one- and two-dimensional (^1^H-^1^H COSY, ^1^H-^13^C HETCOR, ^1^H-^13^C HMBC) spectra. ^1^H and ^13^C as well as 2D NMR spectra of selected **Br-*o*** amide derivative are included in the Supplementary material (Figures S1–S5). Additionally, the analytical signals in the ^1^H and ^13^C NMR spectra as well as the position of characteristic amide I and amide II bands in the FT-IR spectra of all obtained mono-substituted *N-*benzyl amides of **SAL** are collected in Table 1. In Figure 2 the FT-IR spectrum of **SAL-Na** (black dashed line) is compared with that of **SAL** (red dotted line) and that of **Cl-*p*** amide (blue solid line).

The broad bands with the maxima at 3373 cm^−1^ and 3484 cm^−1^ in the spectra of **SAL-Na** and **SAL**, respectively, correspond to the ν(OH) stretching vibration of three OH groups present in these molecules. Different positions of these bands demonstrate the existence of different hydrogen bonds strength within their structures. Additionally, the spectrum of **SAL-Na** shows two intense bands with maxima at 1565 cm^−1^ and 1404 cm^−1^ assigned to ν_as_(COO^−^) and ν_s_(COO^−^) stretching vibrations, respectively. These two bands are absent in the spectrum of **SAL** and instead, a new broad band with a maximum at 1713 cm^−1^ assigned to the superposition of ν(C=O) stretching vibration of both ketone and COOH groups is observed. In the spectrum of **Cl-*p*** three bands most characteristic of this compound are well visible. The narrow band with a maximum at 3038 cm^−1^ corresponds to ν(CH)sp^2^ stretching vibrations, whereas two intense bands with maxima at 1658 cm^−1^ and 1526 cm^−1^are assigned to amide I and amide II, respectively.

In the ^13^C NMR spectra (all performed in CD_2_Cl_2_) of mono-substituted *N-*benzyl amides of **SAL** the characteristic signal of C(1) atom of the amide group is observed in a very narrow range 175.3–176.2 ppm, while the signals of C(1) atom of **SAL-Na** and **SAL** are found at 185.0 ppm and 177.8 ppm, respectively. The characteristic signal of proton of NH_(amide)_ group in the ^1^H NMR spectra of mono-substituted *N-*benzyl amides of **SAL** is in the range of 6.89–7.37 ppm (Table 1).

### 2.2. X-ray Analysis

Structural data of **SAL** amides are very important to explain their biological activity and to perform SAR analysis. Therefore, the structures of three crystalline mono-substituted *N-*benzyl amides (**F-*o***, **F-*m*** and **NO_2_-*o***) were characterized using single crystal X-ray diffraction method. Single crystals of **F-*o*** and **F-*m*** derivatives were obtained by crystallization in the ethanol/water mixture and single crystals of **NO_2_-*o***, in the form of acetonitrile solvates, were grown by crystallization in acetonitrile.

The crystallographic data and structure refinements of **SAL** *N-*benzyl amides **F-*o***, **F-*m*** and **NO_2_-*o*** are summarized in Table S1 (Supplementary Materials).

**Figure 3:**
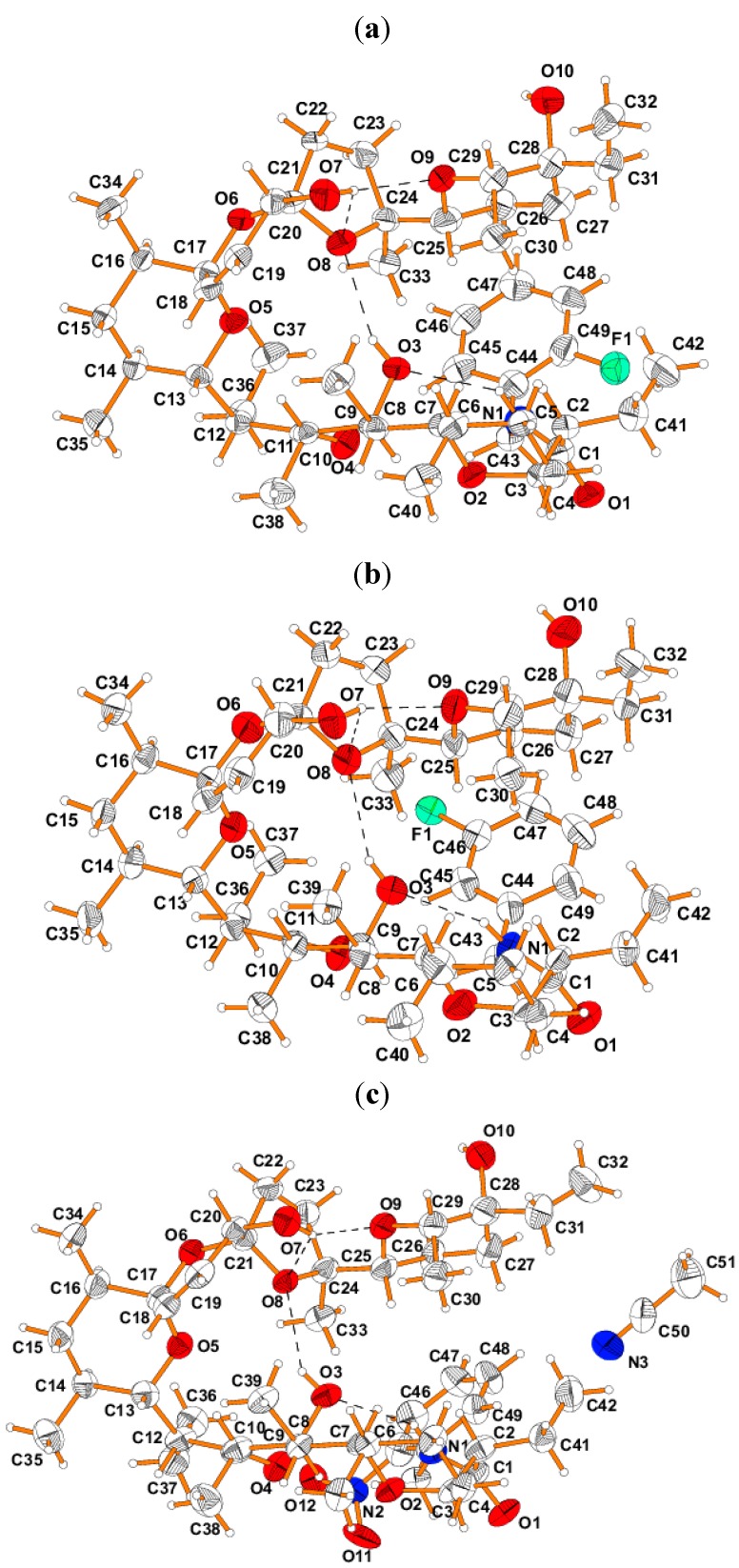
View of the molecular structures of **SAL** amides: (**a**) **F-*o***, (**b**) **F-*m*** and (**c**) **NO_2_-*o***.

Furthermore their molecular structures are presented in Figure 3. The bond lengths and angles characterizing the geometry of the molecules are presented as supplementary material (Table S2). The absolute configuration of **SAL** skeleton of these *N-*benzyl amides is *2R*, *4R*, *6S*, *7R*, *8S*, *9S*, *10S*, *12R*, *13S*, *14S*, *16R*, *17R*, *20R*, *21S*, *24S*, *25R*, *28R* and *29S*, analogously to that found by Kinashi *et al.* [28,29] for the unmodified SAL. The **SAL** skeleton contains four six-membered and one five-membered rings. Two six- and one five-membered rings form tricyclic spiroketal rings system, in which the central ring is unsaturated. The environment of the spiro C17 and C19 heads is tetrahedral, so that the junctions between the five- and six-membered and between both six-membered rings are of the spiro type. The C18-C19 bond of the length of 1.317(2), 1.296(2) and 1.298(2) Å in **SAL** *N-*benzyl amides **F-*o***, **F-*m*** and **NO_2_-*o***, respectively, has a double bond character. The unsaturated six-membered ring with one double bond (C18=C19) in these amides has envelope conformation, where C21 is out of the plane formed by the other ring atoms by 0.553(3), 0.547(3) and 0.599(3) Å in **F-*o***, **F-*m*** and **NO_2_-*o***, respectively. Both saturated six-membered rings in these **SAL** derivatives exhibit chair conformation, and the five-membered ring is twisted with C22 out of the plane of the other ring atoms by 0.384(3), 0.385(3) and 0.441(3) Å in **F-*o***, **F-*m*** and **NO_2_-*o***, respectively. The conformations of the rings as well as the C-C and C-O bonds in these **SAL** derivatives are very similar (Table S2).

The conformation of the **SAL** skeleton of these three *N-*benzyl amides, similar to free **SAL**, exhibits pseudo-cyclic conformation due to the presence of the intramolecular O-H^...^O and N-H^...^O hydrogen bonding interactions. In these *N-*benzyl amides of **SAL**, the OH groups at C9 and C20 atoms are donors of the hydrogen bonds and the NH group of amides also participate as donor forming N1-H^...^O3 hydrogen bonds with similar geometries (Table 2).

**Table 2:** Hydrogen-bond geometry (Å, °) in the crystal structures of **SAL** benzyl amides (**F-*o***, **F-*m*** and **NO_2_-*o***).

D-H···A	D-H	H···A	D···A	D-H···A
**F-*o***				
**O3-H···O8**	0.82 (2)	2.27 (2)	3.016 (2)	152 (2)
**O7-H···O8**	0.82 (2)	2.31 (2)	2.728 (2)	113 (1)
**O7-H···O9**	0.82 (2)	2.26 (2)	3.075 (2)	172 (2)
**O10-H···O1 ^i^**	0.82 (2)	2.27 (2)	3.017 (2)	152 (1)
**N1-H···O3**	0.86 (2)	2.46 (2)	3.166 (2)	139 (2)
**F-*m***				
**O3-H···O8**	0.82 (2)	2.28 (2)	3.013 (2)	150 (2)
**O7-H···O8**	0.82 (2)	2.40 (2)	2.700 (2)	103 (1)
**O7-H···O9**	0.82 (2)	2.36 (2)	3.147 (2)	161 (1)
**O10-H···O1 ^j^**	0.82 (2)	2.17 (2)	2.980 (2)	172 (2)
**N1-H···O3**	0.86 (2)	2.25 (2)	3.048 (2)	155 (2)
**NO_2_-*o***				
**O3-H···O8**	0.82 (2)	2.45 (2)	3.066 (2)	133 (1)
**O7-H···O8**	0.82 (2)	2.26 (2)	2.728 (2)	117 (1)
**O7-H···O9**	0.82 (2)	2.13 (2)	2.873 (2)	151 (1)
**O10-H···O1 ^k^**	0.82 (2)	2.10 (2)	2.789 (2)	141 (1)
**N1-H···O3**	0.86 (2)	2.27 (2)	3.013 (2)	145 (1)

Symmetry code: (i) x+1, y, z; (j) x, y+1, z; (k) x−1, y, z.

Beside these three intramolecular hydrogen bonds, which are more linear than the fourth intramolecular O7-H^...^O8 hydrogen bond, because the OH group at the C20 atom forms a bifurcated hydrogen bond (O7-H^...^O8 and O7-H^...^O9). The oppositely polarized atoms of **SAL** of these benzyl amides are responsible for the folding of the skeleton, and as a result, the intramolecular hydrogen bonds are formed to stabilize the pseudo-cyclic conformation. In this conformation of **SAL** skeleton of these benzyl amides the exterior surface is hydrophobic, and the interior is hydrophilic. In the crystal structure of the compounds studied, the OH group (O10) is at C28 and the carbonyl group (C=O) is at C1, which leads to interactions between the molecules in the crystals. Thus the OH group at C28 is a donor, whereas the carbonyl group at C1 is an acceptor in the intermolecular hydrogen bonds with the neighbors. The intermolecular O-H^...^O hydrogen bonding interactions between the oppositely polarized OH and carbonyl groups, together with the van der Waals forces, are responsible for organization of molecules in the crystals (Figure S6).

### 2.3. ESI MS Evaluation of Ionophoretic Ability

The intracellular and extracellular concentration gradients of metal cations are essential for normal cell functioning [30]. Therefore, the biological properties of **SAL** are related to its ability to complex metal cations and transport them across the lipid membranes from the external environment into the cell. The coordinated ions are released there, which results in a disruption of the natural Na^+^/K^+^ concentration gradient and intracellular pH change, leading to mitochondrial injury, cell swelling, vacuolization and, finally, programmed cell death (apoptosis) [31].

**Figure 4:**
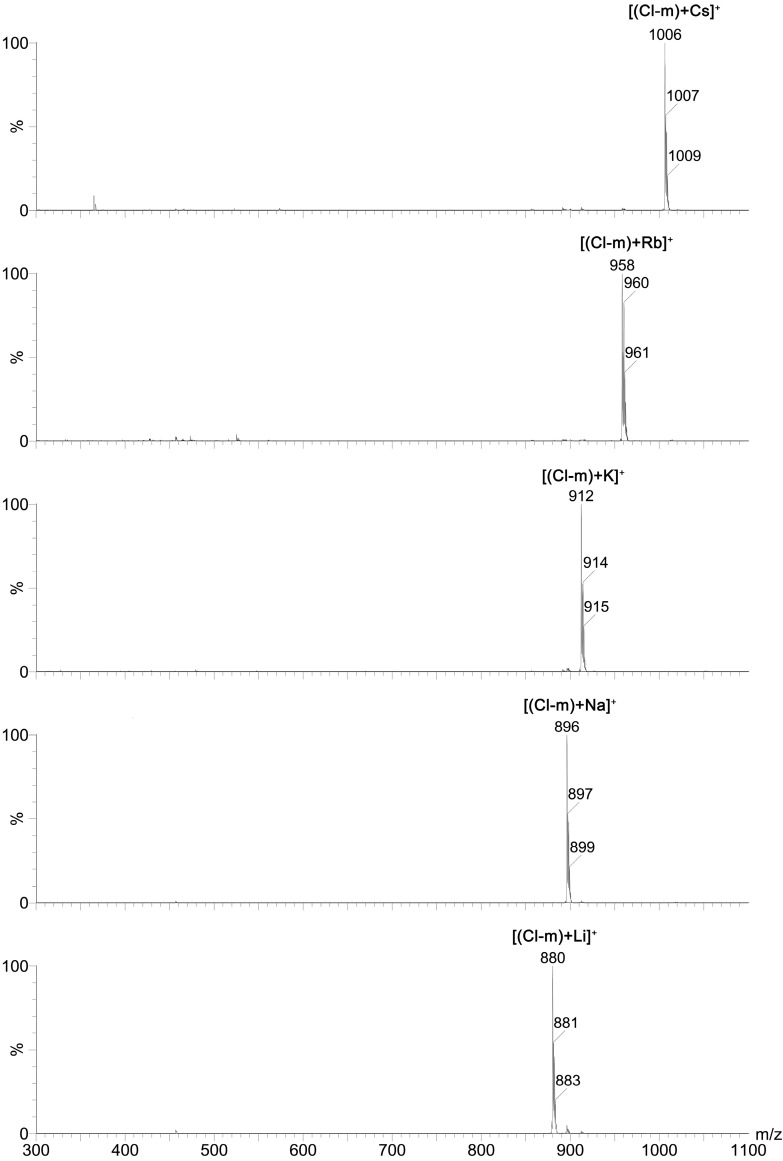
ESI spectra of a mixture of **Cl-*m*** with MClO_4_ (M = Li, Na, K, Rb, Cs) at cv = 30 V.

**Figure 5:**
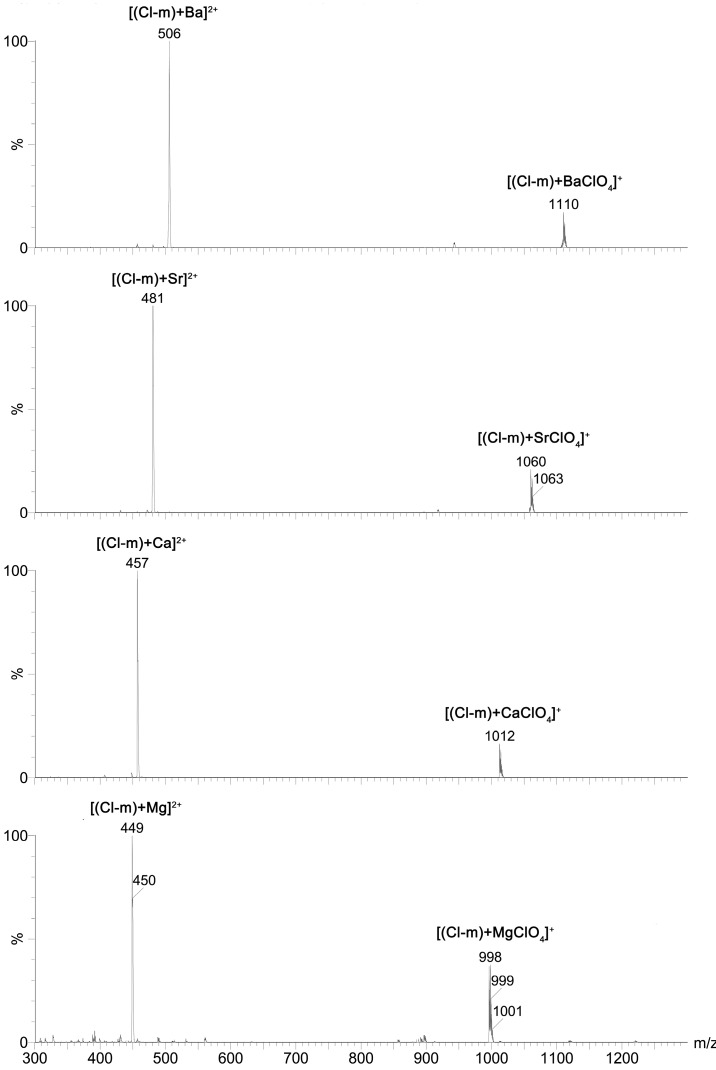
ESI spectra of a mixture of **Cl-*m*** with M(ClO_4_)_2_ (M = Mg, Ca, Sr, Ba) at cv = 30 V.

For this reason, the complexation ability of the new mono-substituted benzyl amides of **SAL** towards monovalent (Li^+^, Na^+^, K^+^, Rb^+^ and Cs^+^) as well as divalent (Mg^2+^, Ca^2+^, Sr^2+^ and Ba^2+^) cations was studied using ESI MS technique. The exemplary ESI MS spectra of **Cl-*m*** complexes with all of these mono- and divalent cations are included in the Figure 4 and Figure 5, respectively.

Results of these studies clearly showed that mono-substituted **SAL** *N-*benzyl amides formed 1:1 complexes with both mono- and divalent metal cations, wherein the chemically unmodified **SAL** formed complexes with monovalent metal cations only. This indicates that the chemical modification of **SAL** has considerable influence on the ionophoretic properties of this antibiotic.

### 2.4. Anticancer in Vitro Activity

The most important aim of the study was to determine the biological activity of the obtained compounds, especially their anticancer activity. MDR of cancer cells is actually one of the greatest problems in the application of chemotherapy and in effective fight against neoplastic diseases. For this reason, all novel mono-substituted *N-*benzyl amides of **SAL** were tested against four human cancer cell lines, including two drug-resistant cell lines.

The tests were performed on promyelocytic leukemia cells and its vincristine-resistant subline (HL-60 and HL-60/vinc, respectively) as well as colon adenocarcinoma cells and its doxorubicin-resistant subline (LoVo and LoVo/dx, respectively). Additionally, the toxicity of these compounds was also tested against normal murine embryonic fibroblasts (BALB/3T3). The reference compounds in these studies were two commonly used anticancer agents—cisplatin and doxorubicin. The concentrations (in µM) of the individual compounds, at which the 50% growth inhibition of cancer cells were observed, are summarized in Table 3.

**Table 3:** Anticancer activity of **SAL** and its monosubstituted *N-*benzyl amides. Data are given as IC_50_ [µM].

Compound	Cancer Cells				Normal Cells	
	HL-60	HL-60/vinc	LoVo	LoVo/dx	BALB/3T3
**SAL**	0.33 ± 0.12	2.33 ± 0.12	0.47 ± 0.08	0.81 ± 0.32	13.02 ± 7.90
**F-*o***	3.63 ± 0.14	4.44 ± 1.73	3.49 ± 0.59	2.32 ± 0.69	5.97 ± 2.07
**F-*m***	64.95 ± 35.32	NA	13.53 ± 0.28	3.91 ± 2.10	6.46 ± 1.11
**F-*p***	3.60 ± 0.69	11.79 ± 2.56	6.18 ± 0.91	3.29 ± 0.91	8.63 ± 1.96
**Cl-*o***	3.18 ± 0.69	3.99 ± 1.47	3.85 ± 0.05	2.91 ± 0.22	6.00 ± 2.26
**Cl-*m***	3.83 ± 0.09	6.59 ± 1.22	11.62 ± 5.12	2.96 ± 0.99	17.34 ± 8.94
**Cl-*p***	36.19 ± 3.03	46.81 ± 12.45	19.27 ± 4.03	16.58 ± 4.81	7.62 ± 1.44
**Br-*o***	3.13 ± 0.71	4.80 ± 1.48	3.64 ± 0.07	2.87 ± 0.29	6.51 ± 3.23
**Br-*m***	3.38 ± 1.09	7.08 ± 1.73	5.23 ± 0.22	3.39 ± 0.15	9.25 ± 1.84
**Br-*p***	20.66 ± 4.66	33.52 ± 10.88	16.29 ± 0.96	4.97 ± 2.76	8.39 ± 2.70
**NO_2_-*o***	3.47 ± 0.44	6.84 ± 2.04	3.81 ± 0.18	2.45 ± 0.26	9.55 ± 2.81
**NO_2_-*m***	8.78 ± 1.73	24.57 ± 8.00	16.15 ± 1.61	3.26 ± 0.84	24.91 ± 2.42
**NO_2_-*p***	NA	NA	13.30 ± 1.94	20.40 ± 8.63	6.91 ± 1.86
**doxorubicin**	0.04 ± 0.04	0.88 ± 0.26	0.15 ± 0.06	5.46 ± 1.56	0.18 ± 0.07
**cisplatin**	1.00 ± 0.23	6.87 ± 1.63	3.70 ± 1.20	5.20 ± 1.93	5.30 ± 2.93

The IC_50_ value is defined as the concentration of a compound that corresponds to a 50% growth inhibition. Human promyelocytic leukemia (HL-60) and its vincristine-resistant subline (HL-60/vinc); human colon adenocarcinoma cell line (LoVo) and doxorubicin resistant subline (LoVo/dx); normal murine embryonic fibroblast cell line (BALB/3T3). Data are expressed as the mean ± SD; NA: not active in concentrations used (up to 100 µg/mL).

As follows from these data, all the compounds tested are more or less active in the specified concentration range depended on the tested cell line. **SAL** derivatives broke (strongly or moderately) the MDR of cancer cells used and this process depended on the chemical nature of **SAL** derivatives. The most active in these tests were chemically unmodified **SAL** (IC_50_ = 0.33–2.33 µM) and its four *N*-benzyl amides: **F-*o***, **Cl-*o***, **Br-*o*** and **NO_2_-*o*** (IC_50_ = 2.32–6.84 µM). Very interesting is the activity of **SAL** derivatives against drug-resistant cell lines, especially against LoVo/dx cancer cell line. This activity in almost all cases was higher than that of the reference compounds, particularly for **F-*o*** and **NO_2_-*o*** (IC_50_ = 2.32 µM and 2.45 µM, respectively). Results of these studies indicate that these derivatives exhibit preferential activity against doxorubicin-resistant colon adenocarcinoma cells. Simultaneously, **SAL** and its derivatives were less toxic (IC_50_ = 5.97–24.91 µM) against normal murine embryonic fibroblasts than commonly used cytostatic drugs—cisplatin and doxorubicin.

For better determination of the ability to MDR breaking and toxicity against normal cells, the indexes of resistance (IR) and selectivity index (SI) values were calculated and are collected in Table 4.

**Table 4:** The calculated values of the indexes of resistance (IR) and selectivity (SI) of **SAL** and its *N-*benzyl amides.

Compound	HL-60	HL-60/vinc		LoVo	LoVo/DX	
	SI	SI	IR	SI	SI	IR
**SAL**	39.45	5.59	7.06	27.70	16.07	1.72
**F-*o***	1.64	1.34	1.22	1.71	2.57	0.66
**F-*m***	0.10	-	-	0.48	1.65	0.29
**F-*p***	2.40	0.73	3.28	1.40	2.62	0.53
**Cl-*o***	1.89	1.50	1.25	1.56	2.06	0.76
**Cl-*m***	4.52	2.63	1.72	1.49	5.86	0.25
**Cl-*p***	0.21	0.16	1.29	0.40	0.46	0.86
**Br-*o***	2.08	1.36	1.53	1.79	2.27	0.79
**Br-*m***	2.74	1.31	2.09	1.77	2.73	0.65
**Br-*p***	0.41	0.25	1.62	0.52	1.69	0.30
**NO_2_-*o***	2.75	1.40	1.97	2.51	3.90	0.64
**NO_2_-*m***	2.84	1.01	2.80	1.54	7.64	0.20
**NO_2_-*p***	-	-	-	0.52	0.34	1.53
**doxorubicin**	4.50	0.20	22.00	1.20	0.03	36.40
**cisplatin**	5.30	0.77	6.87	1.43	1.02	1.41

The IR and SI values were calculated for each compounds using formula: RI = IC_50 _for drug-resistant cell line/IC_50 _for appropriate drug-sensitive cell line; SI = IC_50_ for normal cell line (BALB/3T3)/IC_50_ for respective cancerous cell line.

The IR values indicate how many times a resistant subline is chemo-resistant relative to its parental cell line. When IR is 0–2 the cells are sensitive to the compound tested; IR from the range 2–10 means that the cell shows moderate sensitivity to a given drug; IR above 10 indicates strong drug-resistance. Results of these studies indicated that almost all **SAL** derivatives strongly break MDR of tested cell lines, much stronger than unmodified **SAL**, as well as cisplatin and doxorubicin. This overcoming is especially high for LoVo/dx cell line (IR = 0.20–0.86, except for **NO_2_-*p*** with IR = 1.53), which confirms the high efficiency of derivatives obtained against this cell line.

A beneficial SI > 1.0 indicates a drug with efficacy against cancer cells greater than toxicity against normal cells. In most cases **SAL** derivatives were found more selective against cancer cells than against normal cells of the body. What is interesting, the best results were obtained once again against doxorubicin-resistant colon adenocarcinoma cells (SI = 1.65–7.64, except **Cl-*p*** and **NO_2_-*p***). Additionally, the values of SI coefficient in almost all cases were much higher than for two reference compounds used in the tests.

### 2.5. Antibacterial in Vitro Activity

Chemically unmodified **SAL** and its mono-substituted *N-*benzyl amides were subjected to tests of their antimicrobial activity against different strains of Gram-positive and Gram-negative bacteria, especially against MRSA and MRSE. This activity was evaluated by two parameters: GIZ (Growth Inhibition Zone) and MIC (Minimum Inhibitory Concentration). MRSA strains are resistant to all β-lactams as well as some other and widely used antibiotics. One of the few effective anti-MRSA compounds is ciprofloxacin. Therefore, this compound was used as a reference in the tests.

Among all tested compounds only **SAL** and two amide derivatives **F-*p*** and **NO_2_-*p*** were found active, however, their activity concerned only standard strains of Gram-positive bacteria (Table 5) as well as MRSA and MRSE (Table 6).

**Table 5:** Antibacterial activity of **SAL** as well as its **F-*p*** and **NO_2_-*p*** amides and ciprofloxacin, designated as diameter of GIZ (mm) and MIC (µg/mL).

Bacterial Strains	SAL		F-*p*		NO_2_-*p*		ciprofloxacin	
	GIZ	MIC	GIZ	MIC	GIZ	MIC	GIZ	MIC
*S. aureus* ATCC 4163	30	2	18	32	un	128	26	0.25
*S. aureus* ATCC 25923	29	2	19	32	un	128	26	0.5
*S. aureus* ATCC 6538	34	2	19	16	un	128	28	0.25
*S. aureus* ATCC 29213	28	4	20	32	un	128	22	0.5
*S. epidermidis* ATCC 12228	34	2	20	16	un	128	30	0.25
*S. epidermidis* ATCC 35984	33	2	18	32	un	128	32	0.125

un: unmeasurable.

**Table 6:** Antibacterial activity of **SAL** as well as its **F-*p*** and **NO_2_-*p*** amides against MRSA and MRSE designated as MIC (µg/mL).

*Staphylococcus* Strains	SAL	F-*p*	NO_2_-*p*	Ciprofloxacin
	MIC	MIC	MIC	MIC
**Hospital strains of methicillin resistant *Staphylococcus aureus* (MRSA)**				
452/11	16	16	128	32
456/11	16	16	128	32
462/11	16	32	128	64
514/11	8	32	128	32
522/12	16	32	128	32
537/12	16	32	256	64
572/12	16	32	128	64
573/12	16	64	256	32
585/12	16	32	256	64
586/12	16	64	256	64
**Hospital strains of methicillin-resistant *Staphylococcus epidermidis* (MRSE)**				
459/11	16	32	128	16
460/11	16	32	128	0.125
461/11	16	32	256	0.25
466/11	8	16	256	2
467/11	16	64	128	16
468/11	16	64	128	16
469/11	16	64	256	8
470/11	16	32	256	0.125
488/11	16	128	256	16
489/11	16	32	256	0.25

None of the tested compounds, including unmodified **SAL**, was active against Gram-negative bacteria and fungi of the *Candida* genus (GIZ = 10–12 mm and MIC ≥ 256 µg/mL). This result has been explained by much greater complexity of the structure of Gram-negative bacteria cell wall. The outer membrane of these bacteria is impermeable to hydrophobic compounds, such as ionophores, and their complexes [32].

*Staphylococcus aureus* is a species of bacteria commonly encountered on the skin and/or mucous membranes of the nose of healthy people, which is harmless, but when it gets into the human body can cause serious blood, bone or joints infections. MRSA is the strain of *Staphylococcus aureus* resistant to methicillin and other penicillin type antibiotics [33] and is now a very serious problem in hospitals worldwide. It is noteworthy that the activity of **F-*p*** against MRSA was comparable to the activity of ciprofloxacin and in some cases was even higher than activity of the reference compound (MIC = 16–64 µg/mL and 32–64 µg/mL for **F-*p*** and ciprofloxacin, respectively). Simultaneously, antibacterial activity of **NO_2_-*p*** is much lower than that of **F-*p*** in the tests performed.

### 2.6. Tuberculostatic in Vitro Activity

According to the World Health Organization (WHO), besides HIV/AIDS, tuberculosis is the greatest killer worldwide. This disease, caused by *Mycobacterium tuberculosis* bacteria strains [34], is particularly dangerous for people with weakened immune systems, such as people living with HIV, malnutrition, or diabetes. Simultaneously, very serious problem is the risk of the extensive drug-resistant (XDR) tuberculosis, caused by mycobacteria resistant to at least rifampicin and isoniazid as well as to any member of the quinolone group and at least one of the following second-line anti-TB injectable drugs: kanamycin, capreomycin, or amikacin [35]. Therefore, it is important to search for new drugs, which will be effective, both against sensitive as well as resistant tuberculosis strains.

It is worth noting that there is no information about the antituberculosis properties of **SAL** and its derivatives in the scientific literature. For this reason chemically unmodified antibiotic and its mono-substituted benzyl amides were examined *in vitro* against *M. tuberculosis* standard H_37_R_v_ strain as well as two “wild” strains isolated from tuberculosis patients: one (spec. 210) resistant to *p*-aminosalicylic acid, isonicotinic acid hydrazide, etambutol and rifampicine, and the other (spec. 192) sensitive to the administered antituberculosis drugs (Table 7). The reference compound in the tests was isoniazid.

The tests performed clearly showed that *N-*benzyl amides of **SAL** exhibited low tuberculostatic activity with MIC ranged from 50 µg/mL to more than 100 µg/mL. For comparison, the MIC values were in the range of 25–50 µg/mL and about 3.1–6.25 µg/mL for **SAL** and isoniazid, respectively. Among **SAL** derivatives obtained, the most active in these tests were **F-*p*** and **NO_2_-*p*** amides (MIC = 50 µg/mL against standard and “wild” strains of *M. tuberculosis*). The least active compounds were **Br-*o*** and **Br-*m*** derivatives (MIC > 100 µg/mL in all cases).

**Table 7:** Antitubercular activity of **SAL** and its *N-*benzyl amides. Data are given as MIC [µg/mL].

Compound	*M. tuberculosis* Strains		
	Standard H_37_R_v_ Strain	Isoniazid-Sensitive Strain	Isoniazid-Resistant Strain
**SAL**	25	25	50
**F-*o***	100	50	100
**F-*m***	100	>100	>100
**F-*p***	50	50	50
**Cl-*o***	100	50	>100
**Cl-*m***	100	>100	>100
**Cl-*p***	100	100	>100
**Br-*o***	>100	>100	>100
**Br-*m***	>100	>100	>100
**Br-*p***	50	50	100
**NO_2_-*o***	50	50	100
**NO_2_-*m***	100	100	>100
**NO_2_-*p***	50	50	50
**Isoniazid**	<3.1	<3.1	6.25

### 2.7. Structure-Activity Relationship 

The relationship between the chemical structure of **SAL** derivatives and their biological activity is expected to identify the functional groups, which induce high biological activity and thus to help design more effective chemical modifications of **SAL**. This relation is also vital for the synthesis of compounds, which could be used not only in veterinary medicine, but also for example in human medicine.

Detailed analysis of the obtained data has revealed that the most anticancer active derivatives are those substituted at the -*ortho* position. This rule applies to all tested cell lines, both drug-sensitive as well as drug-resistant cancer cells. Secondly, the least anticancer active are the derivatives substituted at the -*para* position. The exception to this rule is **F-*p***, whose activity is much higher than that of **F-*m*** amide (IC_50_ = 3.60 µM and IC_50_ = 64.95 µM against HL-60 cell line for **F-*p*** and **F-*m***, respectively, as well as IC_50_ = 6.18 µM and IC_50_ = 13.53 µM against LoVo cell line for **F-*p*** and **F-*m***, respectively). Only when tested against LoVo/dx cell line, the activity of these two compounds is high and comparable (IC_50_ = 3.29 µM and IC_50_ = 3.91 µM for **F-*p*** and **F-*m***, respectively). Moreover, the results of anticancer activity studies indicate that all benzyl derivatives, except **Cl-*p*** and **NO_2_-*p***, exhibit preferential activity against LoVo/dx cell line and this activity is higher than that of two commonly cytostatic drugs—cisplatin and doxorubicin (IC_50_ = 2.32–4.97 µM, IC_50_ = 5.20 µM and IC_50_ = 5.46 µM for mono-substituted benzyl amides, cisplatin and doxorubicin, respectively). The least harmful to normal cells are the derivatives substituted at the -*meta* position. This applies to the entire series of these compounds only with one exception—the least cytotoxic compound among fluorinated amides is **F-*p*** (IC_50_ = 5.97 µM, IC_50_ = 6.46 µM and IC_50_ = 8.63 µM for **F-*o***, **F-*m*** as well as **F-*p***, respectively). For all derivatives this activity is lower than that of the reference compounds (IC_50_ = 5.97–24.91 µM, IC_50_ = 5.30 µM and IC_50_ = 0.18 µM for mono-substituted *N-*benzyl amides, cisplatin and doxorubicin, respectively).

Among all amides of **SAL**, only **F-*p*** and **NO_2_-*p*** derivatives showed antimicrobial activity against standard strains of Gram-positive bacteria (MIC = 16–32 µg/mL and MIC = 128 µg/mL for **F-*p*** and **NO_2_-*p***, respectively) as well as MRSA and MRSE (MIC = 16–128 µg/mL and 128–256 µg/mL for **F-*p*** and **NO_2_-*p***, respectively). These great differences in biological activity of the compounds investigated may be connected with the Warburg effect and/or with different mechanisms of ion transport by polyether antibiotics, including **SAL**. Three different ion transport mechanisms through the cell membranes realized by polyether antibiotics are described in literature: (a) electroneutral transport, when the transmembrane potential is maintained; (b) electrogenic transport, when the transmembrane potential is changed and (c) biomimetic transport implemented by polyether antibiotics with the chemically modified COOH group, such as mono-substituted *N-*benzyl amides of **SAL** [19,20].

The Warburg effect is observed in most cancer cells, which predominantly produce energy by a high rate of glycolysis followed by lactic acid fermentation in the cytosol, rather than by a comparatively low rate of glycolysis followed by oxidation of pyruvate in mitochondria. It has been postulated that this change in metabolism is the fundamental cause of cancer diseases [36].

In parallel, high anticancer activity of **SAL** derivatives is probably associated with the mechanism of cations transport realized by such compounds. In cancer cells that are highly acidic, the most common electroneutral transport cannot be effectively carried out, because the COOH group does not undergo deprotonation. Biomimetic transport is then preferred. On the contrary, in bacterial cells the electroneutral or electrogenic transport of ions is preferred and hence much lower antimicrobial than anticancer activity of the **SAL** derivatives tested. This conclusion is confirmed by the fact that the most tuberculostatic active agent is **SAL** and its two *N-*benzyl amides only: **NO_2_-*p*** and **F-*p*** (MIC = 50 µg/mL in all cases). What is interesting, exactly the same derivatives proved to be active in the antibacterial tests. Detailed analysis of anti-tuberculosis activity implies the following conclusions.

Firstly, the most active are the derivatives substituted at the -*para* position. The only exception to this rule is **Cl-*o*** compound, which is about 2-fold more active than the corresponding **Cl-*p*** amide (MIC = 50 µg/mL and MIC = 100 µg/mL against isoniazid-sensitive *M. tuberculosis* strain, respectively).

Secondly, the least tuberculostatic active are the derivatives substituted at the -*meta* position and this applies to the entire series of compounds obtained (MIC > 100 µg/mL in most cases).

Thirdly, the activity against normal H_37_R_v_ strain of all **SAL** *N-*benzyl amides is comparable with that against isoniazid-sensitive strain (MIC = 50–100 µg/mL) and, simultaneously, much higher against isoniazid-resistant strain (MIC > 100 µg/mL in most cases).

## 3. Experimental Section

### 3.1. General

All precursors for the synthesis (amines) and solvents were obtained from Sigma Aldrich (St. Louis, MO, USA) or Fluka (St. Louis, MO, USA) and were used as received without further purification. CD_2_Cl_2_ spectral grade solvent was stored over 3 Å molecular sieves for several days. All manipulations with the substances were performed in a carefully dried and CO_2_-free glove box. TLC was carried out on precoated plates (TLC silica gel 60 F254, Aluminum Plates, Merck (Dormstadt, Germany) and spots were detected by illumination with an UV lamp and visualized with iodine. All the solvent used in flash chromatography were of HPLC grade (CHROMASOLV from Sigma Aldrich) and were used as received. The elemental analysis of **SAL** and its *N-*benzyl amides were carried out on Vario ELIII (Elementar, Hanau, Germany).

### 3.2. Spectroscopic Measurements

The ^1^H and ^13^C spectra were recorded on a Bruker Avance DRX 600 spectrometer (Bruker, Karlsruhe, Gemany). ^1^H NMR measurements of **SAL** and its *N-*benzyl amides (0.07 mol·dm^−3^) in CD_2_Cl_2_ were carried out at the operating frequency 600.055 MHz; flip angle, pw = 45°, spectral width, sw = 4500 Hz; acquisition time, at = 2.0 s; relaxation delay, d_1_ = 1.0 s; T = 293.0 K and using TMS as the internal standard. No window function or zero filling was used. Digital resolution was 0.2 Hz per point. The error of the chemical shift value was 0.01 ppm. The ^13^C NMR spectra were recorded at the operating frequency 150.899 MHz; pw = 60°; sw = 19.000 Hz; at = 1.8 s; d_1_ = 1.0 s; T = 293.0 K and TMS as the internal standard. Line broadening parameters were 0.5 or 1 Hz. The error of chemical shift value was 0.1 ppm. All spectra were locked to deuterium resonance of CD_2_Cl_2_.

The ^1^H and ^13^C NMR signals were assigned using 2D (^1^H-^1^H COSY, ^1^H-^13^C HETCOR, ^1^H-^13^C HMBC) spectra shown in the Supplementary Material. 2D spectra were recorded using standard pulse sequences from Varian and Bruker pulse-sequence libraries.

The FT-IR spectra of **SAL** and its *N-*benzyl amides in the mid infrared region were recorded in KBr. A cell with Si windows and wedge-shaped layers was used to avoid interferences (mean layer thickness 170 mm). The spectra were taken on an IFS 113v FT-IR spectrophotometer (Bruker) equipped with a DTGS detector; resolution 2 cm^−1^, NSS = 64. The HappeGenzel apodization function was used.

The ESI (Electrospray Ionization) mass spectra were recorded on a Waters/Micromass (Manchester, UK) ZQ mass spectrometer equipped with a Harvard Apparatus syringe pump. The samples ware prepared in dry acetonitrile (5 × 10^−5^ mol·dm^−3^). The sample was infused into the ESI source using a Harvard pump at a flow rate of 20 mL·min^−1^. The ESI source potentials were: capillary 3 kV, lens 0.5 kV, extractor 4 V. The standard ESI mass spectra were recorded at the cone voltages: 10 and 30 V. The source temperature was 120 °C and the desolvation temperature was 300 °C. Nitrogen was used as the nebulizing and desolvation gas at flow-rates of 100 dm^3^·h^−1^. Mass spectra were acquired in the positive ion detection mode with unit mass resolution at a step of 1 *m/z* unit. The mass range for ESI experiments was from *m/z* = 300 to *m/z* = 1100.

### 3.3. X-ray Measurement

A colorless single crystal of **F-*o***, **F-*m*** and **NO_2_-*o*** was used for data collection on a four circle KUMA KM-4 diffractometer equipped with a two dimensional CCD detector. The graphite monochromatized MoKα radiation (λ = 0.71073 Å) and the ω-scan technique (Δω = 1.0°) were used for data collection. Data collection and reduction along with the absorption correction were performed using CrysAliss software package [37]. The structure was solved by direct methods using SHELXS-97 program [38] revealing positions of almost all non-hydrogen atoms. The remaining atoms were located from difference Fourier maps. The hydrogen atoms of CH, CH_2_ and CH_3_ groups were constrained with a distance of 0.97 Å and U_iso_ = 1.5 U_eq_ of C joined H. The hydrogen atom of OH groups was also constrained with a distance of 0.82 Å. Visualization of the structure was made with the Diamond 3.1 program [39]. Details on the crystal data, data collection parameters and final refinement parameters are collected in Table S1. Selected geometrical parameters are listed in Table S2. Full details on data collection and refinement, fractional atomic coordinates, anisotropic displacement parameters and full list of bond lengths and angles of the crystal structures of **F-*o***, **F-*m*** and **NO_2_-*o*** in CIF format have been deposited at the Cambridge Crystallographic Data Centre, No. CCDC 1006247, 1006248 and 1006249 for **F-*o***, **F-*m*** and **NO_2_-*o***, respectively. Copies of this information may be obtained free of charge from The Director, CCDC, 12 Union Road, Cambridge, CB2 1EZ, UK (fax: +44-1223-336-033; email: deposit@ccdc.cam.ac.uk or www: http:/www.ccdc.cam.ac.uk).

### 3.4. Synthesis

#### 3.4.1. Isolation of **SAL-Na**

**SAL-Na** was isolated from Sacox^®^120 microGranulate an anticoccidial feed additive distributed by Huvepharma Polska. 100 g of permix was dissolved in dichloromethane. The solvent was evaporated under reduced pressure and the crude obtained product was purified by Dry Vacuum Column Chromatography [27] (gradient solvent mixture hexane/dichloromethane) giving 6 g pure **SAL-Na**. The spectroscopic data of **SAL-Na** were in agreement with previously published assignments [22].

#### 3.4.2. Synthesis of **SAL**

**SAL-Na** was dissolved in dichloromethane and stirred vigorously with a layer of aqueous sulfuric acid (pH = 1.5). The organic layer containing **SAL** was washed with distilled water, and then dichloromethane was evaporated under reduced pressure to dryness giving **SAL**. The spectroscopic data of **SAL** data were in agreement with previously published assignments [22].

#### 3.4.3. General Procedure for the Synthesis of **SAL** Mono-Substituted N-benzyl Amides

To a mixture of **SAL** (500 mg, 0.66 mmol) in dichloromethane (15 mL) the following compounds were added: DCC (206 mg, 1.0 mmol), HOBt (45 mg, 0.33 mmol) and corresponding monosubstituted benzyl amine (2.0 mmol). The mixture was first stirred at a temperature below 0 °C for 6 h and then for further 18 h at room temperature. The solvent was subsequently evaporated under reduced pressure to dryness. The residue was suspended in hexane and filtered off. The filtrate was evaporated under reduced pressure and the residue was purified chromatographically on silica gel (Fluka type 60) to give mono-substituted benzyl amides of SAL (yield from 67% to 84%, see Table 1) as a white solid state. The ^1^H, ^13^C as well as 2D NMR spectra of selected **Br-*o*** amide of **SAL** are included in the Supplementary material.

### 3.5. Antiproliferative Activity of **SAL** and Its Derivatives

Four human cancer cell lines and one normal cell line were used to evaluate anticancer activity of **SAL** and its *N-*benzyl amides: human acute promyelocytic leukemia (HL-60) and its vincristine resistant subline (HL-60/vinc), human colon adenocarcinoma cell lines sensitive (LoVo) and resistant to doxorubicin (LoVo/dx) as well as also normal murine embryonic fibroblast cell line (BALB/3T3). The BALB/3T3 cell line was purchased from the American Type Culture Collection (ATCC Rockville, MD, USA), HL-60 cell line from European Type Culture Collection by courtesy of Professor Spik and Dr Mazurier (Laboratory of Biological Chemistry USTL, Lille, France) and HL-60/vinc, LoVo and LoVo/dx by courtesy of Prof. E. Borowski (Technical University of Gdańsk, Gdańsk, Poland). All the cell lines are maintained in the Institute of Immunology and Experimental Therapy (IIET), Wrocław, Poland.

Human leukemia cells were cultured in ISCOVE medium (IIET, Wroclaw, Polond) containing 10% fetal bovine serum and 2 mM l-glutamine (Sigma Aldrich) and 1 µg/100 mL doxorubicin for HL-60/vinc (Sigma Aldrich). Human colon adenocarcinoma cell lines were cultured in mixture of OptiMEM and RPMI 1640 (1:1) medium (IIET) supplemented with 5% fetal bovine serum (Thermo Fisher Scientific, Waltham, MA, USA), 2 mM l-glutamine, 1 mM sodium pyruvate (Sigma Aldrich) and 10 µg/100 mL doxorubicin for LoVo/dx (Sigma Aldrich). Murine embryonic fibroblast cells were cultured in Dulbecco medium (Gibco, Darmstadt, Germany) supplemented with 10% fetal bovine serum (Thermo Fisher Scientific) and 2 mM l-glutamine (Sigma Aldrich). All culture media contained antibiotics: 100 U/mL penicillin (Sigma Aldrich) and 100 µg/mL streptomycin (Polfa-Tarchomin, City, Poland). All cell lines were cultured during entire experiment in humid atmosphere at 37 °C and 5% CO_2_.

#### 3.5.1. The Anticancer Assays *in Vitro*

Twenty four hours before adding the tested compounds all cell lines were seeded in 96-well plates (Sarstedt, Nümbrecht, Germany) in appropriate media with 10^4^ cells per well. All cell lines were exposed to each tested agent at four different concentrations from the range 100 to 0.1 µg/mL for 72 h. The cells were also exposed to the reference drugs: cisplatin (Accord, London, UK) and doxorubicin hydrochloride (Fluka, St. Louis, MO, USA). Additionally, all cell lines were exposed to DMSO (the solvent used for tested compounds, Sigma Aldrich) at the concentrations corresponding to those in the tested agent dilutions. For adherent cells sulphorodamine B assay was performed and MTT assay for leukemia cells.

#### 3.5.2. SRB

After 72 h of incubation with tested compounds the cells were fixed *in situ* by gently adding of 50 µL per well of cold 50% trichloroacetic acid TCA (Avantor Performance Materials, City, Poland) and were incubated at 4 °C for 1 h. Then the wells were washed four times with water and air-dried. Next, 50 µL of 0.2% solution of sulphorodamine B (Sigma Aldrich) in 1% acetic acid (Avantor Performance Materials) were added to each well and plates were incubated at room temperature for 0.5 h. After incubation time, unbound dye was removed by washing plates four times with 1% acetic acid, whereas the stain bound to cells was solubilized with 150 µL of 10 mM Tris base (Sigma Aldrich). Absorbance of each solution was read at Synergy H4 photometer (BioTek Instruments, Winooski, VT, USA) at 540 nm wavelength.

#### 3.5.3. MTT

Proliferation inhibition of leukemia cells by tested compounds was measured by means of MTT assay. Thus, 20 µL of 3-(4,5-dimethylthiazol-2-yl)-2,5-diphenyl tetrazolium bromide solution (Sigma Aldrich) were added to each well and plates were left in cell incubator for 4 h to allow the cells to metabolize yellow MTT to blue formazan. Then, the lysing mixture consisting of 225 mL dimethylformamide, 67.5 g sodium dodecyl sulfate (both from Sigma Aldrich) and 275 mL of distilled water was added in 80 µL volume per well. Plates were incubated for 24 h for the formazan crystals to be released from cells, dissolved and then absorbance of each well was read at Synergy H4 photometer (BioTek Instruments) at 570 nm wavelength.

Results are presented as mean IC_50_ (concentration of the tested compound that inhibits cell proliferation by 50%) ± standard deviation. IC_50_ values were calculated in Cheburator 1.0.2, Dmitry Nevozhay software for each experiment. Compounds at each concentration were tested in triplicates in single experiment and each experiment was repeated at least three times independently. Results are summarized in Table 3 and Table 4. The IR was defined as the ratio of IC_50_ for a given compound calculated for resistant cell line to that measured for its parental drug sensitive cell line (Table 4).

### 3.6. Antimicrobial Activity of **SAL** and Its Derivatives

Microorganisms used in this study were as follows: Gram-positive cocci: *S. aureus* NCTC 4163, *S. aureus* ATCC 25923, *S. aureus* ATCC 6538, *S. aureus* ATCC 29213, *S. epidermidis* ATCC 12228, *S. epidermidis* ATCC 35984, Gram-negative rods: *E. coli* ATCC 10538, *E. coli* ATCC 25922, *E. coli* NCTC 8196, *P. vulgaris* NCTC 4635, *P. aeruginosa* ATCC 15442, *P. aeruginosa* NCTC 6749, *P. aeruginosa* ATCC 27863, *B. bronchiseptica* ATCC 4617 and yeasts: C. albicans ATCC 10231, *C. albicans* ATCC 90028, *C. parapsilosis* ATCC 22019. The other microorganisms used were obtained from the collection of the Department of Pharmaceutical Microbiology, Medical University of Warsaw, Poland.

Antibacterial activity was examined by the disc-diffusion method under standard conditions using Mueller-Hinton II agar medium (Becton Dickinson, Heidelberg, Germany) according to CLSI (previously NCCLS) guidelines [40]. Antifungal activities were assessed using Muellere Hinton agar + 2% glucose and 0.5 mg/mL Methylene Blue Dye Medium [41].

Sterile filter paper discs (9 mm diameter, Whatman No. 3 chromatography paper) were dripped with tested compound solutions (in EtOH) to load 400 mg of a given compound per disc. Dry discs were placed on the surface of appropriate agar medium. The results (diameter of the growth inhibition zone, GIZ) were read after 18 h of incubation at 35 °C.

Minimal Inhibitory Concentration (MIC) was tested by the twofold serial microdilution method (in 96-well microtiter plates) using Mueller-Hinton Broth medium (Beckton Dickinson) according to CLSI guidelines [42]. The stock solution of a tested agent was prepared in EtOH and diluted in sterile water. Concentrations of tested agents ranged from 0.0625 to 512 µg/mL. The final inoculums of all studied microorganisms were 10^5^ CFU·mL^−1^ (colony forming units per mL). Minimal inhibitory concentrations (the lowest concentration of a tested agent that prevents visible growth of a microorganism) were read after 18 h of incubation at 35 °C.

### 3.7. Tuberculostatic Activity of **SAL** and Its Derivatives

Investigation was performed by the classical test-tube method of successive dilution in Youmans’ modification of Proskauer and Beck’s liquid medium containing 10% of bovine serum [43,44]. Bacterial suspensions were prepared from 14-day-old cultures of slowly growing strains [45,46]. Solutions of the compounds in DMSO were tested. Stock solutions contained 10 mg of compounds in 1 mL. Dilutions (in geometric progression) were prepared in Youmans’ medium. The medium containing no investigated substances and isoniazid as a reference drug was used for comparison. Incubation was performed at a temperature of 37 °C. The MIC values were determined as minimum concentration inhibiting the growth of tested tuberculous strains in relation to the probe with no tested compound. The influence of the compound on the growth of bacteria at certain concentrations 3.1, 6.2, 12.5, 25, 50 and 100 µg/mL was evaluated.

## 4. Conclusions

Twelve novel mono-substituted *N-*benzyl amides of **SAL** containing fluorine, chlorine, bromine and nitro substituents at the -*ortho*, -*meta* and -*para* positions were synthesised and characterized by spectroscopic and spectrometric methods. The anticancer activities against two drug-sensitive and two drug-resistant cancer cell lines, as well as antibacterial activities of these amides were tested to get the structure-activity relationships. The most active, except one compound, were *N-*benzyl amides of **SAL** substituted in -*ortho* position and the least anticancer active derivatives were those substituted at the -*para* position. Furthermore, almost all **SAL** *N-*benzyl amides were proved less toxic against normal cells than the commonly used cytostatic agents—cisplatin and doxorubicin. On the other hand, only two of the compounds tested **F-*p*** and **NO_2_-*p*** were active against standard strains of Gram-positive bacteria as well as MRSA and MRSE hospital strains. Additionally, the antitubercular activity of **SAL** derivatives (especially substituted at the -*para* position) was determined for the first time. Further optimizations of **SAL** derivatives should be carried out to discover those having potent higher anticancer activity against human cancer cell lines.

## Supplementary Materials

Supplementary materials can be accessed at: http://www.mdpi.com/1420-3049/19/12/19435/s1.
