# Supplementary material for: Synthesis, Anticancer and Antibacterial Activity of Salinomycin N-Benzyl Amides
Source: Molecules. 2014 Nov 25;19(12):19435–59. doi: 10.3390/molecules191219435 (PMC6271077; doi:10.3390/molecules191219435)
Supplement: Supplementary file 1 [file molecules-19-19435-s001.pdf]

Supplementary Materials

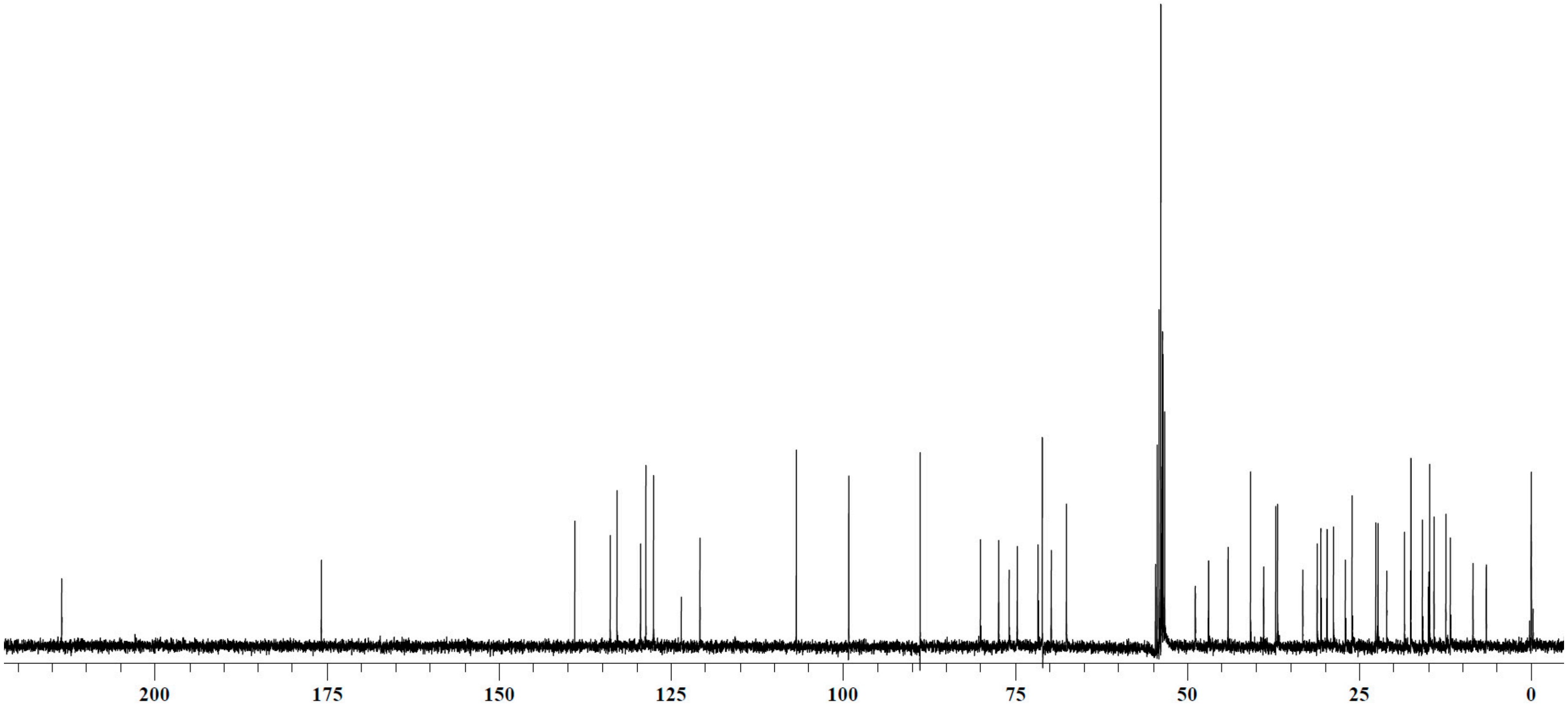

Figure S1. The ^{13}C NMR spectrum of ***Br-o*** in CD_2Cl_2.

List of signals: ^{13}C-NMR (400 MHz, CD_2Cl_2) δ (ppm) 213.5, 175.8, 138.9, 133.8, 132.8, 129.3, 128.6, 127.5, 123.4, 120.7, 106.7, 99.1, 88.7, 79.9, 77.3, 75.8, 74.6, 71.6, 70.9, 69.7, 67.5, 48.7, 46.8, 44.0, 40.7, 38.8, 37.0, 36.8, 33.1, 31.0, 30.5, 29.6, 28.7, 26.9, 25.9, 25.1, 22.5, 22.2, 20.9, 18.3, 17.4, 15.7, 14.8, 14.7, 14.1, 12.3, 11.7, 8.4, 6.5.

Figure S2. The ^{1}H NMR spectrum of ***Br-o*** in CD_2Cl_2.

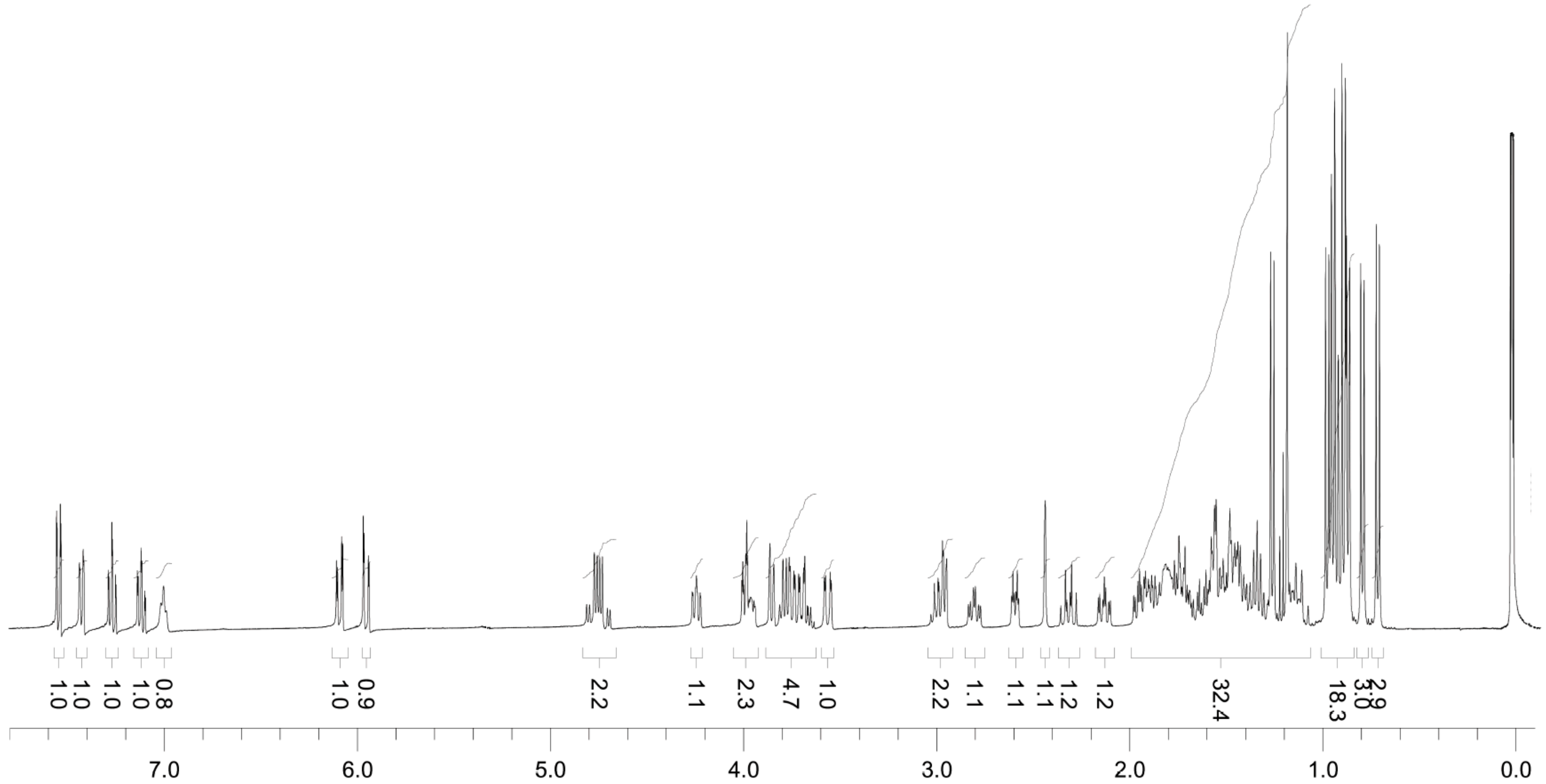

List of signals: ^{1}H-NMR (400 MHz, CD_2Cl_2) δ (ppm) 7.53 (dd, *J* = 7.93, 1.21 Hz, 1H), 7.41 (dd, *J* = 7.69, 1.40 Hz, 1H), 7.25 (dt, *J* = 7.53, 1.21 Hz, 1H), 7.10 (dt, *J* = 7.64, 1.67 Hz, 1H), 6.98 (t, *J* = 5.60 Hz, 1H), 6.07 (dd, *J* = 10.74, 2.23 Hz, 1H), 5.93 (dd, *J* = 10.69, 1.51 Hz, 1H), 4.73 (dq, *J* = 16.20, 5.91 Hz, 2H), 4.25-4.19 (m, 1H), 4.02-3.89 (m, 2H), 3.87-3.60 (m, 5H), 3.54 (dd, *J* = 12.14, 2.45 Hz, 1H), 3.04-2.90 (m, 2H), 2.78 (dt, *J* = 10.79, 4.37 Hz, 1H), 2.57 (td, *J* = 9.06, 2.63 Hz, 1H), 2.42 (s, 1H), 2.30 (td, *J* = 12.61, 9.81 Hz, 1H), 2.11 (ddd, *J* = 12.79, 9.76, 3.22 Hz, 1H), 1.99-1.04 (m, 32H), 1.01-0.81 (m, 18H), 0.77 (d, *J* = 6.92 Hz, 3H), 0.69 (d, *J* = 6.76 Hz, 3H).

Figure S3. The ^{1}H-^{13}C HETCOR spectrum of ***Br-o*** in CD_2Cl_2.

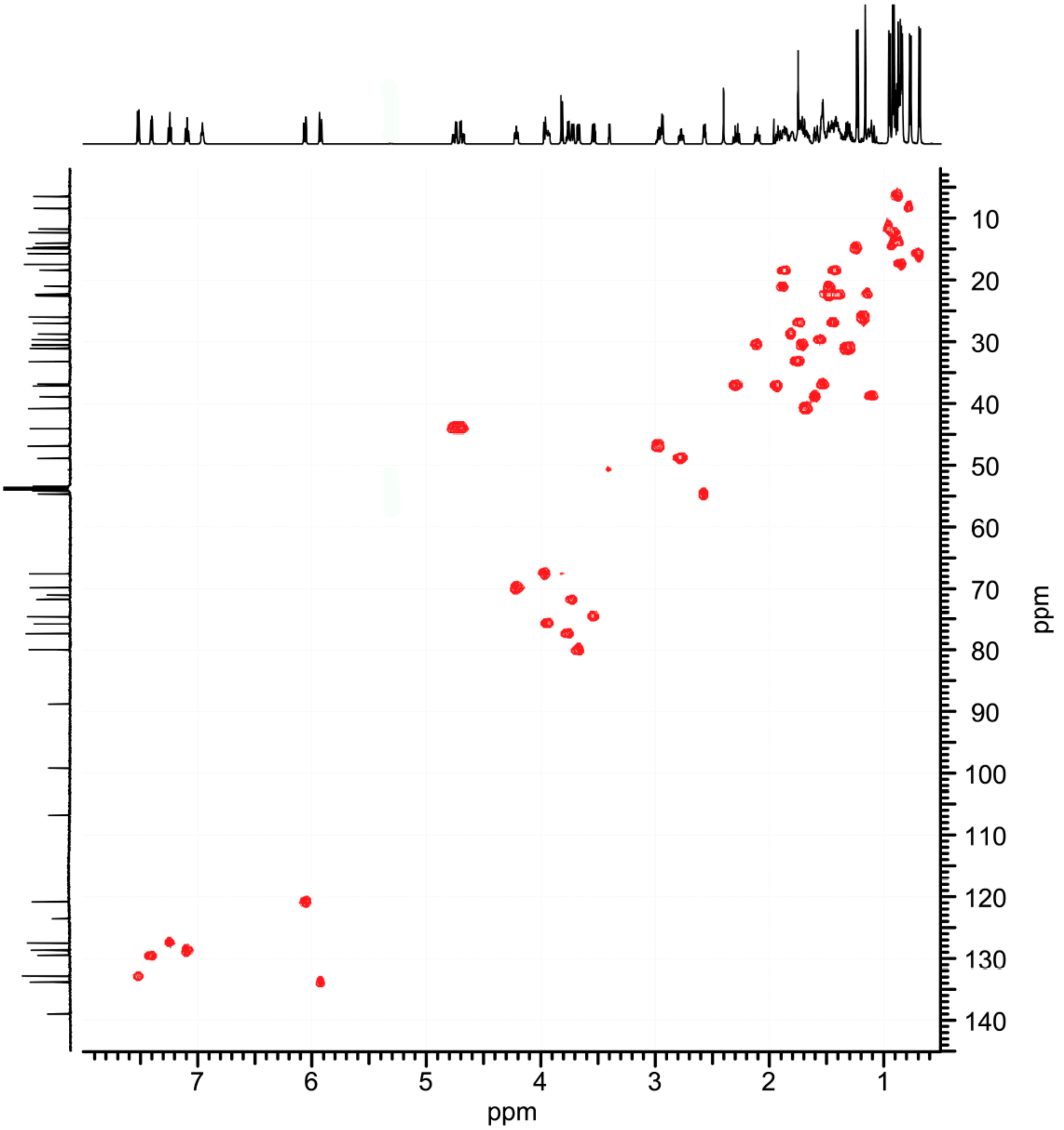

Figure S4. The ^{1}H-^{1}H COSY spectrum of ***Br-o*** in CD_2Cl_2.

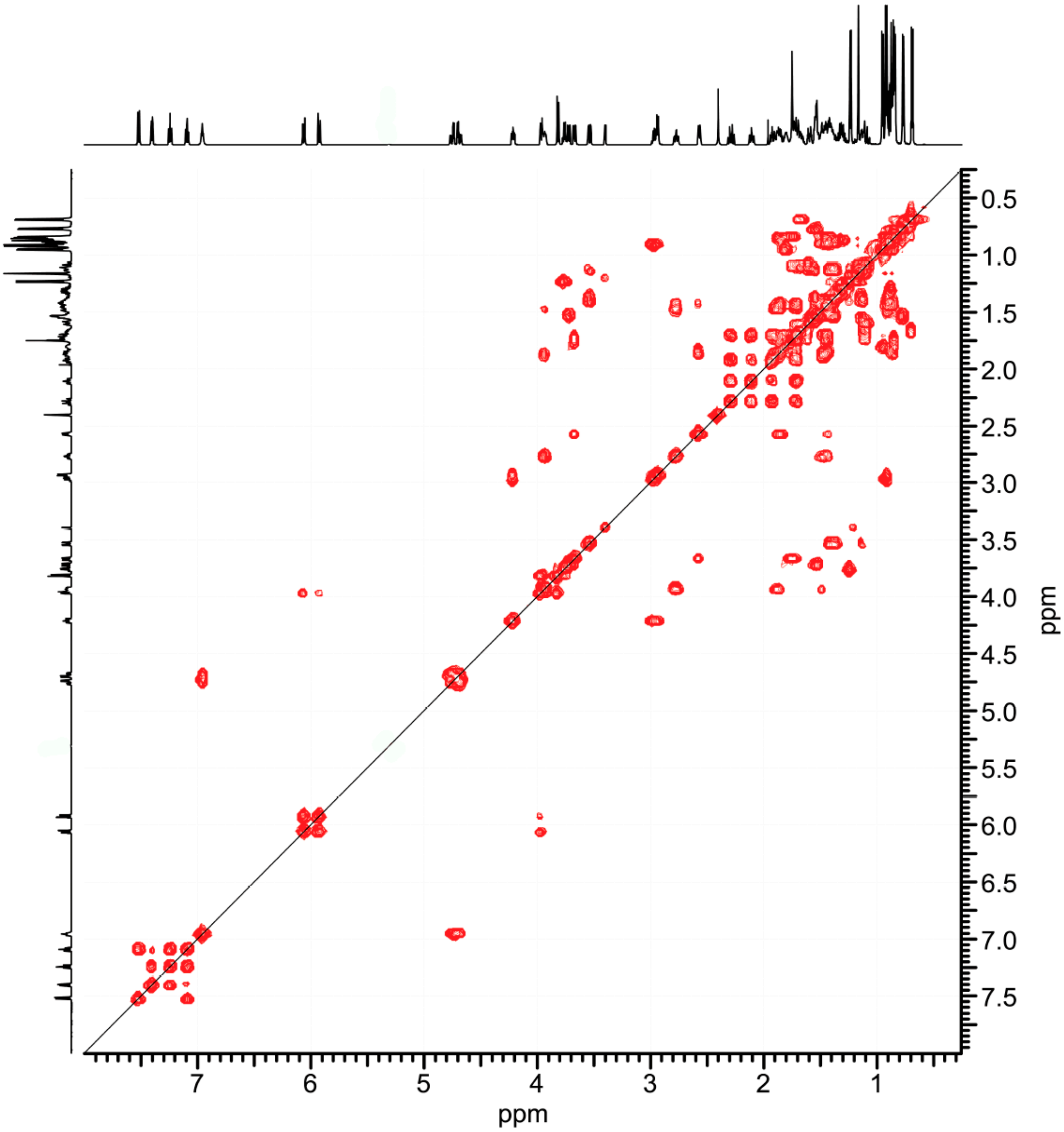

Figure S5. The ^{1}H-^{13}C HMBC spectrum of ***Br-o*** in CD_2Cl_2.

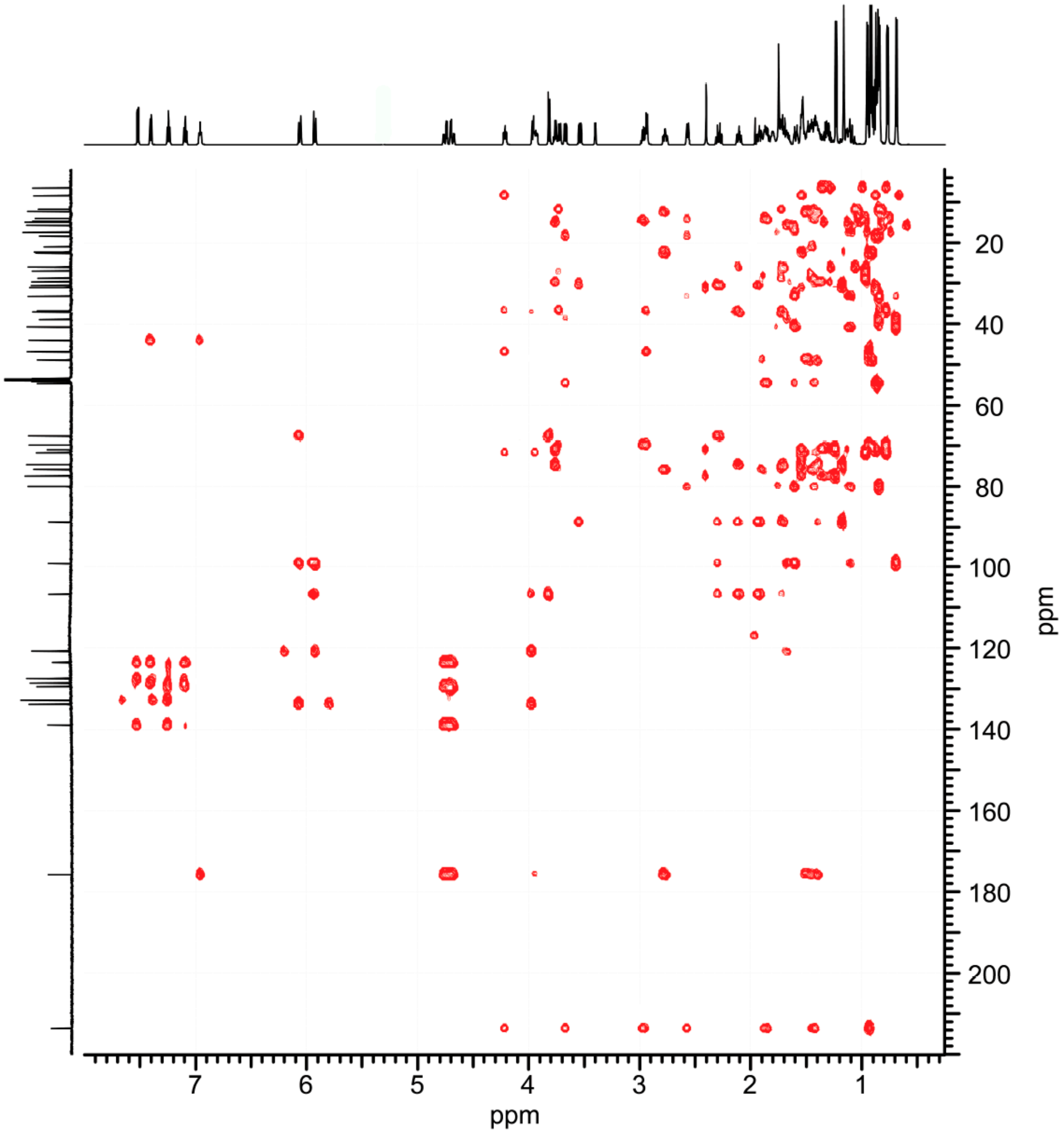

Figure S6. Arrangement of molecules in the crystals showing the interomolecular O-H···O hydrogen bonds between the hydroxyl group at C28 and carbonyl group C1=O: (**a**) **F-*o***, (**b**) **F-*m*** and (**c**) **NO_2-*o***. The figures 6 and 7 are deleted because they are in manuscript. Thus Figures are re-numbered.

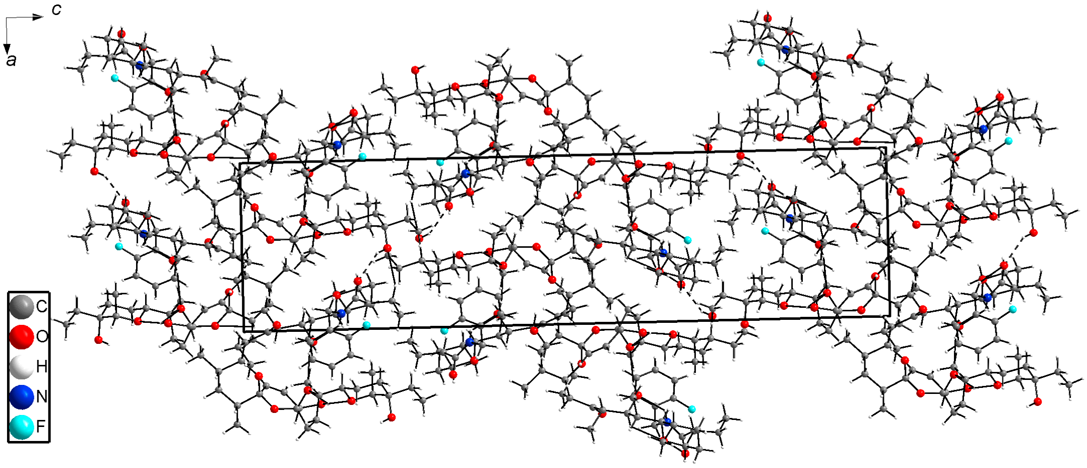

(**a**) **F-*o***

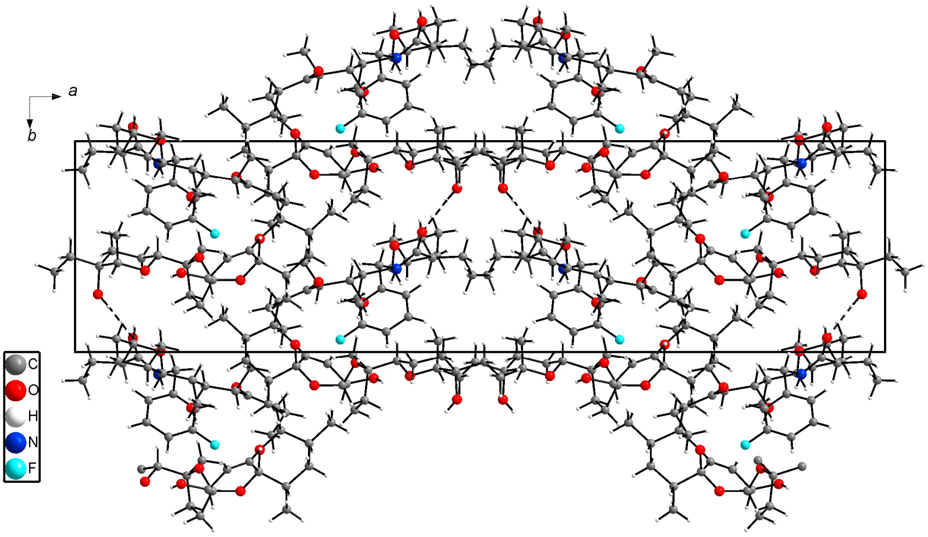

(**b**) **F-*m***

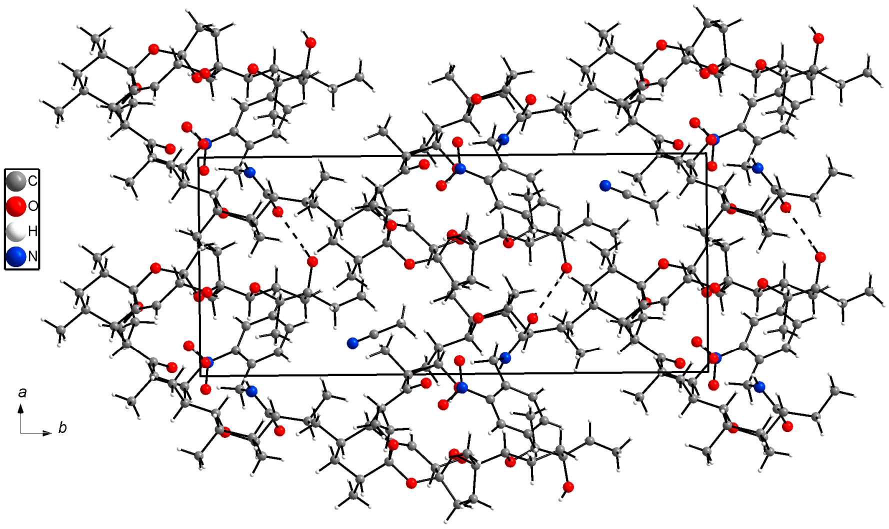

(**c**) **NO_2-*o***

Table S1. Crystal data and structures refinements of SAL benzyl amides (**F-*o***, **F-*m*** and **NO_2-*o***).

	**F-*o***	**F-*m***	**NO_2-*o***
Formula	$C_{49}H_{76}FNO_{10}$	$C_{49}H_{76}FNO_{10}$	$C_{49}H_{76}N_2O_{12}\cdot C_2H_3N$
Formula weight	858.11	858.11	926.17
Crystal system	orthorhombic	monoclinic	monoclinic
Space group	$P2_12_12_1$	$C2$	$P2_1$
a [Å]	10.4157(7)	40.261(4)	10.4527(8)
b [Å]	11.6486(9)	10.402(2)	23.149(2)
c [Å]	39.663(3)	11.756(2)	11.2104(9)
β [°]		96.10(1)	107.30(1)
V [Å^3]	4812.2(6)	4895.5(13)	2589.9(4)
Z	4	4	2
D_{calc}/D_{obs} (g·cm^{-3})	1.184/1.18	1.164/1.16	1.188/1.185
μ (mm^{-1})	0.084	0.082	0.084
F(000)	1864	1864	1004
Crystal size (mm)	0.33 × 0.28 × 0.22	0.35 × 0.28 × 0.23	0.41 × 0.33 × 0.25
MoK_α λ [Å]	0.71073	0.71073	0.71073
Temperature [K]	295(2)	295(2)	295(2)
θ range [°]	2.62–28.41	2.53–28.39	3.25–28.16
T_{min}/T_{max}	0.9766/0.9844	0.9766/0.9878	0.9733/0.9846
Refs collected/unique/observed	51949/11207/5623	26571/9924/5411	24987/11248/6413
R_{int}	0.0398	0.0727	0.0245
R[F_2>2σ(F_2)] [a]	0.0772	0.0743	0.0552
wR (F^2 all reflections) [b]	0.1014	0.1544	0.1281
S	1.002	1.000	1.002
Flack parameter	−0.12 (6)	−0.15(8)	0.18(9)
$\Delta\rho_{max}$, $\Delta\rho_{min}$ (e Å^{-3})	+0.139, −0.130	+0.390, −0.207	+ 0.314, −0.199

[a] $R = \sum||F_o| - |F_c||/\sum F_o$; [b] $wR = \{\sum[w(F_o^2 - F_c^2)^2]/\sum wF_o^4\}^{1/2}$; $w = 1/[\sigma^2(F_o^2) + (aP)^2]$, where $P = (F_o^2 + 2F_c^2)/3$ and a = 0.0250, for structure F-*o*; a = 0.0560, for structure F-*m* and a = 0.0320 for structure NO_2-o.

Table S2. Selected geometrical parameters (Å, °) of **SAL** benzyl amides **F-*o***, **F-*m*** and **NO_2-*o***.

	F-*o*	F-*m*	NO_2-*o*
**C1-O1**	1.219(2)	1.205(2)	1.240(2)
**C1-N1**	1.361(2)	1.350(2)	1.340(2)
**C1-C2**	1.509(2)	1.515(3)	1.500(2)
**C2-C3**	1.535(2)	1.531(3)	1.555(2)
**C2-C41**	1.542(2)	1.519(2)	1.519(2)
**C3-O2**	1.436(2)	1.440(2)	1.444(2)
**C3-C4**	1.518(2)	1.513(3)	1.500(2)
**O2-C7**	1.429(2)	1.407(2)	1.443(2)
**C4-C5**	1.478(2)	1.458(3)	1.486(3)
**C5-C6**	1.533(2)	1.555(3)	1.548(3)
**C6-C7**	1.519(2)	1.542(2)	1.524(2)
**C6-C40**	1.539(2)	1.516(3)	1.524(3)
**C7-C8**	1.527(2)	1.519(2)	1.521(2)
**C8-C39**	1.516(2)	1.521(2)	1.544(2)
**C8-C9**	1.545(2)	1.550(2)	1.534(2)
**C9-O3**	1.434(2)	1.425(2)	1.431(2)
**C9-C10**	1.530(2)	1.531(2)	1.526(2)
**C10-C11**	1.519(2)	1.534(2)	1.517(2)
**C10-C38**	1.537(2)	1.529(2)	1.531(2)
**C11-O4**	1.199(2)	1.204(2)	1.204(2)
**C11-C12**	1.543(2)	1.528(2)	1.533(2)
**C12-C36**	1.518(2)	1.535(2)	1.544(3)
**C12-C13**	1.543(2)	1.537(2)	1.537(2)
**C13-O5**	1.438(2)	1.434(2)	1.438(2)
**C13-C14**	1.532(2)	1.535(2)	1.537(2)
**O5-C17**	1.455(2)	1.438(2)	1.438(2)
**C14-C35**	1.520(2)	1.525(2)	1.514(2)
**C14-C15**	1.521(2)	1.517(2)	1.514(2)
**C15-C16**	1.526(2)	1.523(2)	1.525(2)
**C16-C34**	1.522(2)	1.504(2)	1.491(2)
**C16-C17**	1.540(2)	1.537(2)	1.542(2)
**C17-O6**	1.416(2)	1.411(2)	1.417(2)
**C17-C18**	1.476(2)	1.499(2)	1.508(2)
**O6-C21**	1.428(2)	1.423(2)	1.428(2)
**C18-C19**	1.317(2)	1.296(2)	1.298(2)
**C19-C20**	1.492(2)	1.484(2)	1.479(2)
**C20-O7**	1.420(2)	1.417(2)	1.416(2)
**C20-C21**	1.513(2)	1.534(2)	1.522(2)
**C21-O8**	1.421(2)	1.407(2)	1.413(2)
**C21-C22**	1.519(2)	1.505(2)	1.522(2)
**O8-C24**	1.448(2)	1.478(2)	1.463(2)
**C22-C23**	1.489(2)	1.515(3)	1.509(2)

Table S2. *Cont.*

	F-*o*	F-*m*	NO_2-*o*
**C23-C24**	1.550(2)	1.505(2)	1.540(2)
**C24-C25**	1.506(2)	1.509(2)	1.536(2)
**C24-C33**	1.519(2)	1.496(2)	1.500(2)
**C25-O9**	1.422(2)	1.439(2)	1.426(2)
**C25-C26**	1.514(2)	1.513(2)	1.501(2)
**O9-C29**	1.435(2)	1.431(2)	1.441(2)
**C26-C27**	1.521(2)	1.509(3)	1.548(3)
**C27-C28**	1.519(2)	1.507(3)	1.537(4)
**C28-O10**	1.432(2)	1.428(2)	1.429(2)
**C28-C29**	1.519(2)	1.544(3)	1.496(3)
**C28-C31**	1.532(2)	1.530(3)	1.542(3)
**C29-C30**	1.540(2)	1.523(3)	1.505(2)
**C31-C32**	1.508(3)	1.473(3)	1.339(4)
**C36-C37**	1.509(2)	1.475(3)	1.403(4)
**C41-C42**	1.500(2)	1.460(4)	1.516(3)
**N1-C43**	1.443(2)	1.445(2)	1.442(2)
**O1-C1-N1**	120.29(15)	122.00(18)	120.65(13)
**O1-C1-C2**	123.15(14)	120.78(17)	121.18(12)
**N1-C1-C2**	116.53(13)	117.16(16)	118.14(13)
**C1-C2-C3**	107.39(12)	106.60(14)	105.47(12)
**O2-C3-C4**	110.88(13)	109.98(17)	110.63(12)
**O2-C3-C2**	109.07(11)	110.15(14)	109.58(10)
**C2-C3-C4**	118.12(12)	118.81(16)	118.92(13)
**C7-O2-C3**	114.39(10)	115.52(12)	115.15(11)
**C3-C4-C5**	111.62(13)	112.41(18)	112.16(13)
**C4-C5-C6**	110.62(13)	110.79(18)	110.84(16)
**C5-C6-C7**	107.85(13)	106.87(17)	106.59(14)
**O2-C7-C6**	110.64(10)	109.64(14)	109.24(11)
**O2-C7-C8**	106.05(10)	107.09(12)	106.55(11)
**C6-C7-C8**	116.50(12)	116.09(15)	116.56(13)
**C7-C8-C9**	110.34(11)	110.74(13)	111.38(12)
**C8-C9-C10**	113.26(10)	113.08(12)	114.12(12)
**O3-C9-C8**	110.60(10)	109.99(11)	111.09(11)
**O3-C9-C10**	110.35(10)	111.22(11)	110.87(9)
**C9-C10-C11**	113.28(11)	112.29(12)	113.96(12)
**C10-C11-C12**	118.40(11)	117.48(12)	119.31(13)
**O4-C11-C10**	121.33(12)	121.28(13)	120.38(12)
**O4-C11-C12**	119.94(12)	120.93(13)	119.97(12)
**C11-C12-C13**	112.78(10)	117.48(12)	119.31(13)
**C12-C13-C14**	115.35(11)	115.53(12)	116.43(12)
**O5-C13-C12**	106.21(10)	106.71(12)	104.25(12)
**O5-C13-C14**	112.81(10)	112.41(12)	112.02(11)
**C13-O5-C17**	115.03(9)	115.60(11)	115.89(10)
**C13-C14-C15**	107.49(11)	107.08(12)	106.01(12)
**C14-C15-C16**	111.96(11)	112.99(13)	114.33(13)

Table S2. *Cont.*

	F-*o*	F-*m*	NO_2-*o*
**C15-C16-C17**	111.07(10)	110.94(12)	110.86(11)
**O6-C17-O5**	105.13(10)	106.30(11)	104.54(10)
**O6-C17-C18**	112.50(11)	111.61(12)	111.77(11)
**O5-C17-C18**	110.80(10)	110.53(12)	110.90(10)
**O6-C17-C16**	104.88(9)	105.26(11)	104.78(9)
**O5-C17-C16**	108.75(10)	109.82(11)	110.67(11)
**C16-C17-C18**	114.23(11)	113.00(13)	113.65(11)
**C17-O6-C21**	120.87(10)	121.91(11)	119.87(8)
**C17-C18-C19**	124.05(13)	123.41(15)	122.43(13)
**C18-C19-C20**	121.52(14)	123.44(15)	123.54(12)
**O7-C20-C19**	110.94(12)	111.66(15)	112.46 (11)
**O7-C20-C21**	112.48(12)	111.63(13)	111.60(12)
**C19-C20-C21**	111.04(12)	110.28(14)	110.24(12)
**O8-C21-O6**	111.32(10)	111.78(12)	112.23(11)
**O8-C21-C20**	108.71(11)	107.93(13)	108.17(10)
**O6-C21-C20**	110.98(11)	110.51(13)	109.55(11)
**O8-C21-C22**	104.01(11)	106.15(13)	105.46(11)
**O6-C21-C22**	107.43(11)	107.05(13)	105.88(10)
**C20-C21-C22**	114.22(12)	113.39 (14)	115.57(12)
**C21-C22-C23**	106.89(11)	104.24 (14)	104.79(12)
**C21-O8-C24**	112.89(10)	111.14 (11)	112.14(9)
**C22-C23-C24**	104.29(12)	106.49 (15)	104.25(11)
**C23-C24-C25**	114.03(11)	114.18 (14)	114.22(12)
**O8-C24-C25**	106.86(11)	105.66 (12)	105.59(10)
**C24-C25-C26**	116.36(12)	115.46 (14)	115.84(13)
**O9-C25-C26**	110.23(11)	109.98 (14)	108.58(14)
**C25-O9-C29**	114.66(10)	115.06 (13)	114.76(12)
**C25-C26-C27**	110.49(13)	111.06 (15)	110.74(17)
**C26-C27-C28**	111.79(13)	113.81 (16)	113.40(18)
**C27-C28-C29**	110.15(12)	108.81 (15)	109.25(17)
**O10-C28-C27**	109.13(13)	110.05 (16)	110.90(17)
**C28-C29-C30**	114.92(12)	115.32 (16)	115.77(16)
**O9-C29-C28**	111.03(13)	111.46 (16)	112.58(14)
